# IgA Deficiency Exacerbates an Animal Model of Multiple Sclerosis Induced with Theiler’s Virus

**DOI:** 10.3390/cells15181665

**Published:** 2026-09-15

**Authors:** Fumitaka Sato, Alfredo A. Hinay Jr., Seiichi Omura, Ijaz Ahmad, Sandesh Rimal, Cong Thanh Nguyen, Ah-Mee Park, Reona Shiro, Sachiyo Tsuji-Kawahara, Ikuo Tsunoda

**Affiliations:** 1 Department of Microbiology, Faculty of Medicine, Kindai University, Sakai 590-0197, Osaka, Japan; 2 Department of Science and Technology on Food Safety, Faculty of Biology-Oriented Science and Technology, Kindai University, Kinokawa 649-6493, Wakayama, Japan; 3 Department of Tropical Viral Vaccine Development, Institute of Tropical Medicine (NEKKEN), Nagasaki University, Nagasaki 852-8523, Nagasaki, Japan; 4 Department of Arts and Sciences, Faculty of Medicine, Kindai University, Sakai 590-0197, Osaka, Japan; 5 Department of Molecular Genetics & Microbiology, University of New Mexico School of Medicine, Albuquerque, NM 87131, USA; rshiro@salud.unm.edu; 6 Department of Immunology, Faculty of Medicine, Kindai University, Sakai 590-0197, Osaka, Japan

**Keywords:** animal models, bioinformatics, CNS demyelinating diseases, dysbiosis, neuropathology, persistent virus infections, *Picornaviridae* infections

## Abstract

Multiple sclerosis (MS) is an immune-mediated disease characterized by inflammatory demyelination and axonal degeneration in the central nervous system (CNS) and has been linked to viral infections. Theiler’s murine encephalomyelitis virus (TMEV) infection in SJL/J mice has been widely used as a viral model of MS, since TMEV-infected mice develop MS-like demyelination and axonal degeneration with viral persistence in the CNS. In the TMEV model, we previously demonstrated upregulation of immunoglobulin (Ig) A-related genes in the CNS, as well as IgA deposition and IgA-positive cell infiltration in demyelinating lesions. Although IgA has been implicated in the pathophysiology of MS, its precise role remains unclear. To determine the role of IgA in the TMEV model, we established IgA knockout (KO) mice on the SJL/J background; IgA KO mice lacked serum IgA and exhibited altered gut microbiota compositions. We found that TMEV-infected IgA KO mice developed more severe neurological signs than wild-type (WT) mice. IgA KO mice also had more severe inflammatory demyelination with higher levels of viral persistence and T cell infiltration in the CNS than WT mice. Immunologically, IgA KO mice mounted TMEV-specific lymphoproliferative responses and serum anti-viral antibody isotype titers, except for IgA, which were comparable to WT mice. Interleukin (IL)-17 and IL-10 productions were higher in IgA KO mice than in WT mice. Thus, IgA could play a protective role in the TMEV model in WT mice; IgA deficiency-mediated gut dysbiosis may contribute to enhanced immunopathology in IgA KO mice.

## 1. Introduction

Multiple sclerosis (MS) is an immune-mediated disease characterized by inflammatory demyelination and axonal degeneration in the central nervous system (CNS) [1,2]. Although the precise pathomechanism of MS remains unclear, it has been proposed that MS is triggered by the interaction between dysregulated immune responses and viral infection [3]. The immune etiology of MS has been supported by various clinical findings. For example, MS patients have immune cell infiltration and antibody depositions in CNS lesions [4]; higher levels of autoreactive immune responses to the CNS have been detected in MS patients compared with controls [5]. Immunomodulatory drugs, such as anti-very late antigen (VLA)-4 and anti-CD20 monoclonal antibodies, have been effective for MS therapy [6,7,8].

The viral etiology of MS has also been supported clinically. For example, at the protein or gene level, virus-infected cells, such as Epstein–Barr virus (EBV)-infected B cells and human herpesvirus 6 (HHV6)-infected oligodendrocytes, have been detected in the brain of MS patients [9,10]. Following primary EBV infection, the risk of MS has been reported to increase more than 30-fold [11]. Higher anti-viral immune responses have also been detected in MS patients compared with controls [12]. Experimentally, several neurotropic viruses, such as murine hepatitis virus and Sindbis virus, can induce MS-like neuropathology in animals [13,14].

Among these viruses, Theiler’s murine encephalomyelitis virus (TMEV) infection has been widely used as a viral model of MS [15,16]. TMEV is a non-enveloped, positive-sense, single-stranded RNA virus belonging to the family *Picornaviridae* [17]. Intracerebral inoculation of TMEV in susceptible mouse strains, such as SJL/J mice, can cause inflammatory demyelination and axonal degeneration with viral persistence in the CNS about 1-month post-infection (p.i.) (chronic phase). TMEV-induced demyelinating disease (TMEV-IDD) in SJL/J mice has two pathogenic mechanisms: (1) direct lytic viral infection in the CNS (viral pathology) and (2) immune-mediated CNS tissue damage (immunopathology) [18,19,20]. Both humoral and cellular anti-viral immune responses have been shown to play protective and detrimental roles in the TMEV model. Although CD4^+^ T helper (Th) cells and CD8^+^ cytotoxic T lymphocytes (CTL) are crucial to eradicate the virus from the brain during the acute phase of TMEV infection around 1 to 2 weeks p.i., Th17 cells can contribute to the development of viral persistence and inflammatory demyelination in the CNS during the chronic phase [21,22]. Similarly, anti-TMEV antibodies have been known to neutralize the virus, contributing to viral clearance [18]; anti-TMEV antibodies can cross-react with myelin antigens, acting as autoantibodies, exacerbating demyelination [23].

The physiological role of immunoglobulin (Ig) A is homeostasis of mucosal tissues; following IgA production, IgA is transported to the mucosal surface in a dimeric form with the joining (J) chain, neutralizing extracellular pathogens, such as viruses and bacteria [24]. In the CNS, we demonstrated that TMEV-infected SJL/J mice had upregulations of IgA-related genes, including *Igha* and *Jchain*, and IgA depositions and IgA-positive (^+^) cells in demyelinating lesions [25]. Anti-viral IgA responses in cerebrospinal fluid (CSF) have been detected in experimental animals following CNS viral infections, including Sindbis virus and herpes simplex virus (HSV) 1 [26,27]. Clinically, in CNS infections with viruses, such as HSV and varicella-zoster virus, antigen-specific IgA antibodies have been observed in CSF from the patients [28].

IgA has also been shown to regulate the compositions of the gut microbiota [29]. Changes in the gut microbiota compositions, called dysbiosis, have been linked to dysregulated immune responses [30]; IgA and IgA-producing cells have been proposed to play a role in immune-mediated diseases, including inflammatory bowel disease (IBD) and MS [31,32,33]. In MS, the frequencies of IgA-bound bacteria in fecal samples were lower in active MS patients than in inactive MS patients [32]. In patients with MS, IgA concentrations have been shown to increase in CSF [33,34]; IgA-secreting cells infiltrating the CNS have been proposed to originate from the gut and cross-react with several gut microbiota [33]. Furthermore, a subset of *IGHA*-expressing cells with co-expression of *IL10* was detected in active demyelinating lesions [33]. Similarly, in its autoimmune model, experimental autoimmune encephalomyelitis (EAE), IgA-secreting plasma cells were observed in demyelinating lesions; IgA-secreting plasma cells have been proposed to play a protective role by producing an anti-inflammatory cytokine, interleukin (IL)-10 [32].

In this study, we established IgA knockout (KO) mice on the SJL/J background to investigate whether IgA deficiency could affect the TMEV model. IgA KO mice lacked serum IgA and had distinct gut microbiota compositions. After TMEV infection, IgA KO mice developed more severe neurological signs with greater demyelination, viral persistence, and T-cell infiltration in the CNS than wild-type (WT) mice. IL-17 and IL-10 concentrations were higher in TMEV-infected IgA KO mice than in WT mice, although anti-viral T-cell, IgG1, and IgG2c responses, but not IgA, were similar between the two mouse substrains. These findings suggest that IgA may play a protective role in the TMEV model by reducing viral persistence in the CNS and dysbiosis in the gut.

## 2. Materials and Methods

### 2.1. Animal Experiment

Female 4- to 5-week-old SJL/J mice (JAX^®^ Mice Strain) were purchased from the Jackson Laboratory Japan, Inc. (Kanagawa, Japan). IgA KO mice on the SJL/J background were custom-generated at the Laboratory Animal Resource Center, University of Tsukuba (Ibaraki, Japan), using CRISPR/Cas9-mediated genome editing, as described previously [35]. Two guide RNAs (gRNAs) targeting the mouse *Igha* locus were designed. A mixture containing the gRNAs, a modified Cas9 [35], and donor DNA (Supplementary Text S1) was microinjected into the cytoplasm of pronuclear-stage SJL/J zygotes. The viable embryos were subsequently transferred into the oviducts of pseudopregnant female mice. The resulting offspring were genotyped to identify mice carrying the targeted *Igha* allele using the following primer pair: forward, CGGGCTGCAGGAATTCTAGGCTGGACCAAGTTAGGCTGGATGG and reverse, CAAACCTCCCTATGATGCTGACCCCACA. IgA deficiency was further confirmed by serum antibody isotype enzyme-linked immunosorbent assays (ELISAs), as described below. Mice were maintained under specific pathogen-free conditions in the animal care facility at Kindai University Faculty of Medicine (Osaka, Japan). All experimental procedures were reviewed and approved by the Institutional Animal Care and Use Committee of Kindai University Faculty of Medicine (KAME-2022-001 and KAME-2025-010) and were performed in accordance with the criteria outlined by the National Institutes of Health (NIH).

We inoculated 2 × 10^5^ plaque-forming units (PFUs) of the Daniels (DA) strain of TMEV intracerebrally (i.c.) into 5- to 6-week-old WT and IgA KO mice and observed them for 2 months [36]. The neurological signs in TMEV-infected mice were monitored by scoring the severity of the impairment of the righting reflex as follows: the proximal end of the mouse’s tail was grasped and twisted to the right and then to the left: 0, a healthy mouse resists being turned over; 1, the mouse is flipped onto its back but immediately rights itself on one side; 1.5, the mouse is flipped onto its back but immediately rights itself on both sides; 2, the mouse rights itself in 1 to 5 seconds (s); 3, righting takes more than 5 s; and 4, the mouse cannot right itself [15]. Two independent experiments were conducted using four to six mice per group per experiment.

### 2.2. ELISA

To quantify antibody isotype concentrations in serum, blood was collected from naïve WT and IgA KO mice by submandibular bleeding and then centrifuged at 2775× *g* for 20 min at 4 °C for harvesting serum. The serum samples were stored at –80 °C until analyzed, as described previously [37]. We coated 96-well flat-bottom Nunc-Immuno plates (Thermo Fisher Scientific Inc., Waltham, MA, USA) with 20 ng/well of an anti-mouse IgA (SouthernBiotech, Birmingham, AL, USA), IgM (SouthernBiotech), IgG1 (SouthernBiotech), or IgG2c (SouthernBiotech) antibody as the capture antibody. The serum samples were diluted with assay diluent by serial 10-fold dilutions from 10^4^ to 10^6^-fold and added to the plates. Mouse IgA (SouthernBiotech), IgM (SouthernBiotech), IgG1 (Thermo Fisher Scientific Inc.), and IgG2c (GeneTex, Inc., Irvine, CA, USA) were used for generating each standard curve. We added a peroxidase-conjugated anti-mouse F(ab′)_2_ antibody (Jackson ImmunoResearch Laboratories Inc., West Grove, PA, USA) as the detection antibody, followed by the TMB Substrate Reagent Set (BD Biosciences, San Jose, CA, USA) to detect the immunoreactive complexes. The absorbance was measured at 450 nm using the Synergy H1 Hybrid Multi-Mode Microplate Reader (Agilent Technologies, Inc., Santa Clara, CA, USA).

Serum anti-TMEV antibody isotypes were titrated, as described previously [38]. We coated the 96-well plates with 500 ng/well of a TMEV antigen solution. Serum samples were serially diluted by two-fold from 2^7^ to 2^28^-fold and added to the plates. A peroxidase-conjugated anti-mouse IgA (Thermo Fisher Scientific Inc.), IgM (Enzo Biochem Inc., Farmingdale, NY, USA), IgG1 (Thermo Fisher Scientific Inc.), or IgG2c (Thermo Fisher Scientific Inc.) antibody was used as the secondary antibody in each anti-TMEV antibody isotype ELISA. The absorbance higher than the mean + two standard deviations of naïve serum samples at a 2^7^-fold dilution was used as the lower detection limit for evaluating anti-TMEV antibody titers.

### 2.3. Hematoxylin and Eosin Stain

Naïve IgA KO mice were killed with isoflurane (Viatris Inc., Canonsburg, PA, USA) and perfused with phosphate-buffered saline (PBS), followed by a 4% para-formaldehyde (PFA, FUJIFILM Wako Pure Chemical Corporation, Osaka, Japan) solution in PBS. For histological analysis, general organs, including the thymus, heart, spleen, liver, kidney, and intestine, were harvested and embedded in paraffin. We prepared 4-μm-thick tissue sections using the Leica SM2000 R Sliding Microtome (Leica Biosystems, Tokyo, Japan) and stained them with hematoxylin (Electron Microscopy Sciences, Hatfield, PA, USA) and eosin (Thermo Fisher Scientific Inc.).

### 2.4. Microbiome Analysis

Feces were collected from naïve WT and IgA KO mice and stored at −80 °C until analyzed [39]. DNA was purified from the fecal samples using the QIAamp Fast DNA Stool Mini Kit (QIAGEN, Venlo, The Netherlands) according to the manufacturer’s instructions. We performed 16S rRNA amplicon sequencing of the DNA samples (Bioengineering Lab. Co., Ltd., Kanagawa, Japan). Briefly, bacterial DNA was amplified by two-step tailed polymerase chain reaction (PCR) using primer sets for the V3 and V4 regions of bacterial 16S rRNA (Table 1). The bacterial DNA library was processed and sequenced on the NextSeq 1000 System (Illumina, Inc., San Diego, CA, USA). Using QIIME 2, we processed and visualized the raw FASTQ files. The FASTQ files and processed data were deposited in the Sequence Read Archive (SRA) at the National Center for Biotechnology Information (NCBI) (Accession number, PRJNA1510292; URL, https://www.ncbi.nlm.nih.gov/bioproject/PRJNA1510292/, accessed on 10 August 2026).

To compare the richness, evenness, and combination of bacterial richness/evenness between WT and IgA KO mice, we calculated the Faith’s phylogenetic diversity (PD), Pielou’s evenness, and Shannon index, respectively, using QIIME 2, as described previously [39]. We compared the overall gut microbiome between WT and IgA KO mice using principal component analysis (PCA) with an R program “prcomp.” The PCA plot with 80% confidence ellipses for each group was drawn using R version 4.5.2 and the packages “ggplot2” and “dplyr.” A taxonomic bar plot was generated using QIIME 2.

### 2.5. Luxol Fast Blue Stain

TMEV-infected mice were killed with isoflurane 2 months p.i. and perfused with PBS, followed by a 4% PFA solution in PBS, as described previously [40]. We harvested the spinal cord and brain, divided them into 12 to 13 transverse segments and five coronal slabs, respectively, and embedded them in paraffin. We prepared 4-μm-thick CNS tissue sections, using the Leica SM2000 R Sliding Microtome, and stained them with Luxol fast blue (Solvent blue 38; MP Biomedicals, LLC, Irvine, CA, USA) for myelin visualization, followed by cresyl violet (MP Biomedicals, LLC) as a counterstain. To quantify spinal cord pathology scores, we divided each spinal cord section into four quadrants: the ventral funiculus, each lateral funiculus, and the dorsal funiculus [15]. Any quadrant containing demyelination, meningitis, or perivascular cuffing (inflammation) was assigned a score of 1 in the pathological class. The total number of positive quadrants for each pathological class was divided by the total number of quadrants present on the slide and multiplied by 100 to determine the percentage of involvement for each pathological class. Brain pathology scores were determined by combining the pathology scores of meningitis, perivascular cuffing, and demyelination. These scores were evaluated as follows: meningitis: 0, no meningitis; 1, mild cellular infiltration; 2, moderate cellular infiltration; and 3, severe cellular infiltration; perivascular cuffing: 0, no cuffing; 1, 1 to 10 lesions; 2, 11 to 20 lesions; 3, 21 to 30 lesions; 4, 31 to 40 lesions; and 5, over 40 lesions; demyelination: 0, no demyelination; 1, mild demyelination; 2, moderate demyelination; and 3, severe demyelination [41]. Two independent experiments were conducted, with four to six mice per group.

### 2.6. Immunohistochemistry

We visualized IgA, TMEV antigen^+^ cells, T cells, and damaged axons by immunohistochemistry with antibodies against IgA (SouthernBiotech) [25], TMEV [42], CD3 (Biocare Medical, LLC, Pacheco, CA, USA) [41], and non-phosphorylated neurofilaments (SMI 311, an antibody cocktail composed of SMI 32, 33, 37, 38, and 39, BioLegend, San Diego, CA, USA), respectively [15]. For the antibodies to CD3 and SMI 311, the spinal cord sections were pre-treated with the Dako Target Retrieval Solution, Citrate pH 6 (Agilent Technologies, Inc.) or Milli-Q water (Merck KGaA, Darmstadt, Germany), respectively, at 120 °C for 15 min in an autoclave for antigen retrieval. The antigen/antibody complexes were visualized using a peroxidase-conjugated anti-goat antibody (Agilent Technologies, Inc.) or Histofine MAX-PO kits (Nichirei Biosciences Inc., Tokyo, Japan), followed by a 3,3′-diaminobenzidine (DAB, FUJIFILM Wako Pure Chemical Corporation) substrate solution. To quantify TMEV antigen^+^ cells, T cells, and damaged axons, we divided each spinal cord section into four quadrants: the ventral funiculus, each lateral funiculus, and the dorsal funiculus. The numbers of TMEV antigen^+^ cells, T cells, and damaged axons in each quadrant were counted under a light microscope, using 12 to 13 transverse spinal cord segments per mouse, as we described previously [15,21].

### 2.7. Lymphoproliferative Assay

We harvested the spleen from TMEV-infected mice and isolated mononuclear cells (MNCs) using Histopaque^®^-1083 (Merck KGaA) [36]. MNCs were cultured at 2 × 10^5^ cells/well in 96-well flat-bottom microplates (Sumitomo Bakelite Co., Ltd., Tokyo, Japan) and stimulated with or without live TMEV at a multiplicity of infection (MOI) of 5 at 37 °C with 5% CO_2_ for 5 days to assess lymphoproliferative responses to TMEV. We added 3 µL/well of a Cell Counting Kit-8 (CCK-8) solution (Dojindo Laboratories, Kumamoto, Japan) during the last 24 h of culture. We performed all cultures in triplicate and conducted two independent experiments using four to six mice per group per experiment. The absorbance was measured at 450 nm using the Synergy H1 Hybrid Multi-Mode Microplate Reader. Data were expressed as stimulation indices: (mean absorbance with TMEV stimulation)/(mean absorbance without stimulation).

### 2.8. Cytokine ELISA

Splenic MNCs from TMEV-infected mice were cultured at 8 × 10^6^ cells/well in 6-well plates (Sumitomo Bakelite Co., Ltd.) and stimulated with TMEV at an MOI of 1 or with 5 μg/mL of concanavalin A (ConA, Merck KGaA) for 2 days [36]. The culture supernatants were harvested and stored at –80 °C until examined. The concentrations of interferon (IFN)-γ (BD Biosciences), IL-17A (BioLegend), IL-4 (BD Biosciences), and IL-10 (BD Biosciences) in the culture supernatants were quantified in duplicate, using ELISA kits according to the manufacturers’ instructions. The lower detection limits were as follows: IFN-γ, 31.3 pg/mL; IL-17A, 15.6 pg/mL; IL-4, 7.8 pg/mL; and IL-10, 31.3 pg/mL.

### 2.9. Statistical Analysis

Using OriginPro 2026 (OriginLab Corporation, Northampton, MA, USA), Student’s *t*-test and Mann–Whitney *U* test were conducted for parametric and nonparametric data, respectively [15]. When the *p* value was less than 0.05, we considered it a significant difference between WT and IgA KO mice.

## 3. Results

### 3.1. Generation of IgA KO Mice on the SJL/J Background

**Figure 1:**
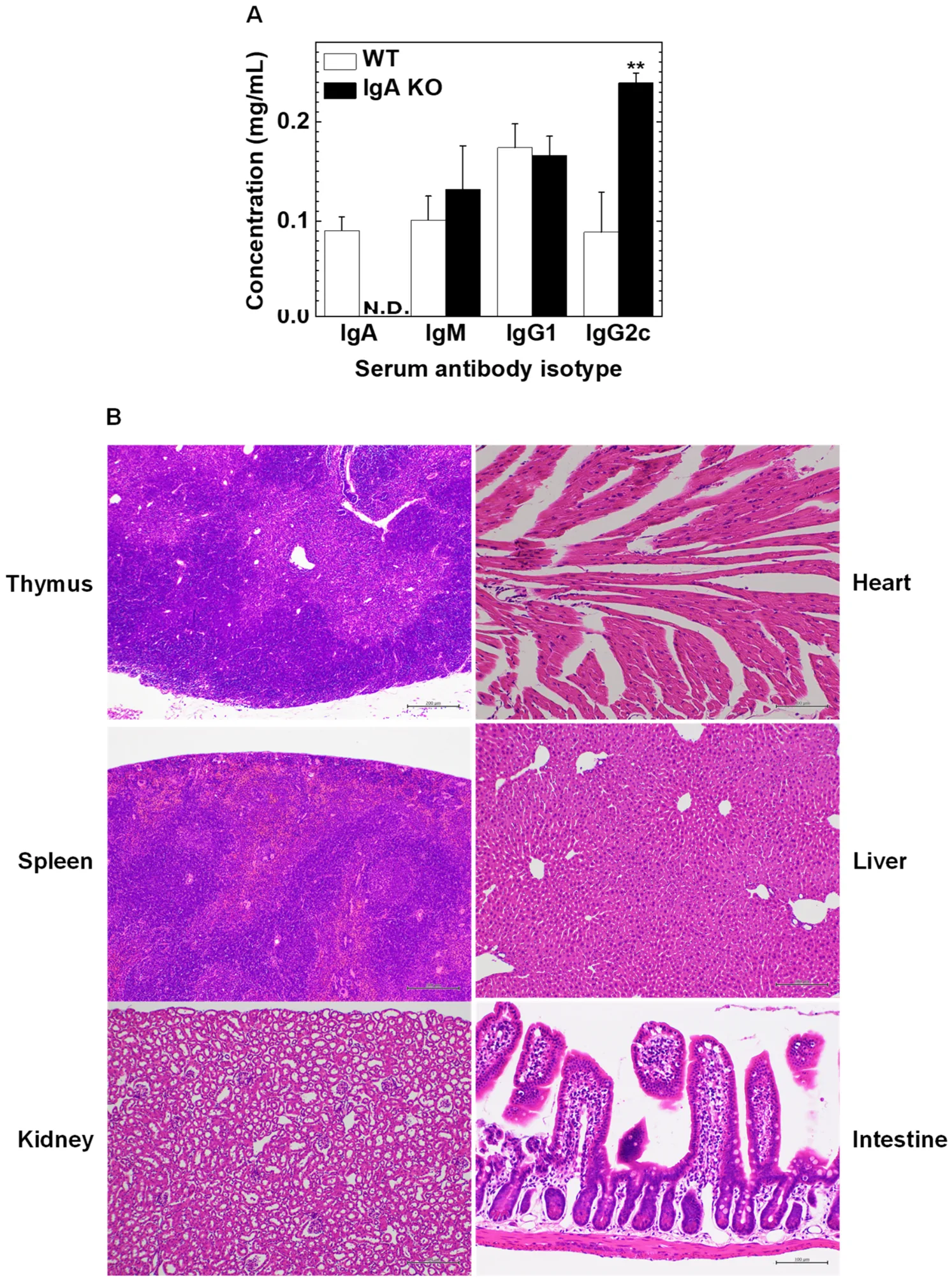
Immunoglobulin (Ig) A knockout (KO) mice on the SJL/J background had normal serum IgM and IgG1, but increased IgG2c, responses and no pathological changes in general organs. (**A**) Serum concentrations of whole antibody isotypes were quantified by enzyme-linked immunosorbent assays (ELISAs). Serum IgA responses were mounted in naïve wild-type (WT) mice (white bar), but not detectable in naïve IgA KO mice (black bar). Serum IgM and IgG1 concentrations were comparable between WT and IgA KO mice; IgA KO mice had significantly higher IgG2c concentrations than WT mice. Values are the mean + standard error of the mean (SEM) for four to five mice/group. ** *p* < 0.01, Student’s *t*-test. (**B**) General organs were stained with hematoxylin and eosin for histological analysis. IgA KO mice exhibited normal pathology in the thymus, heart, spleen, liver, kidney, and intestine. Tissue sections are representative of three naïve IgA KO mice. Scale bar = 100 μm (heart and intestine) and 200 μm (thymus, spleen, kidney, and liver).

IgA KO mice on the SJL/J background were generated as described in the Materials and Methods. To confirm the absence of IgA at the protein level, we quantified serum antibody isotype concentrations in WT and IgA KO mice by ELISAs and found no serum IgA in IgA KO mice (Figure 1A). We measured body weight in 4- to 5-week-old female WT and IgA KO mice and found that IgA KO mice were significantly smaller than age-matched WT mice [mean body weight (g) ± standard error of the mean (SEM): WT (n = 10), 18.4 ± 0.6; IgA KO (n = 9), 16.5 ± 0.6; *p* < 0.05, Student’s *t*-test]. We also conducted histological analysis of IgA KO mice and found normal histological architecture in the thymus, spleen, heart, liver, kidney, and intestine, with no pathological changes (Figure 1B).

### 3.2. IgA KO Mice Have Different Gut Microbiota Compositions from WT Mice

**Figure 2:**
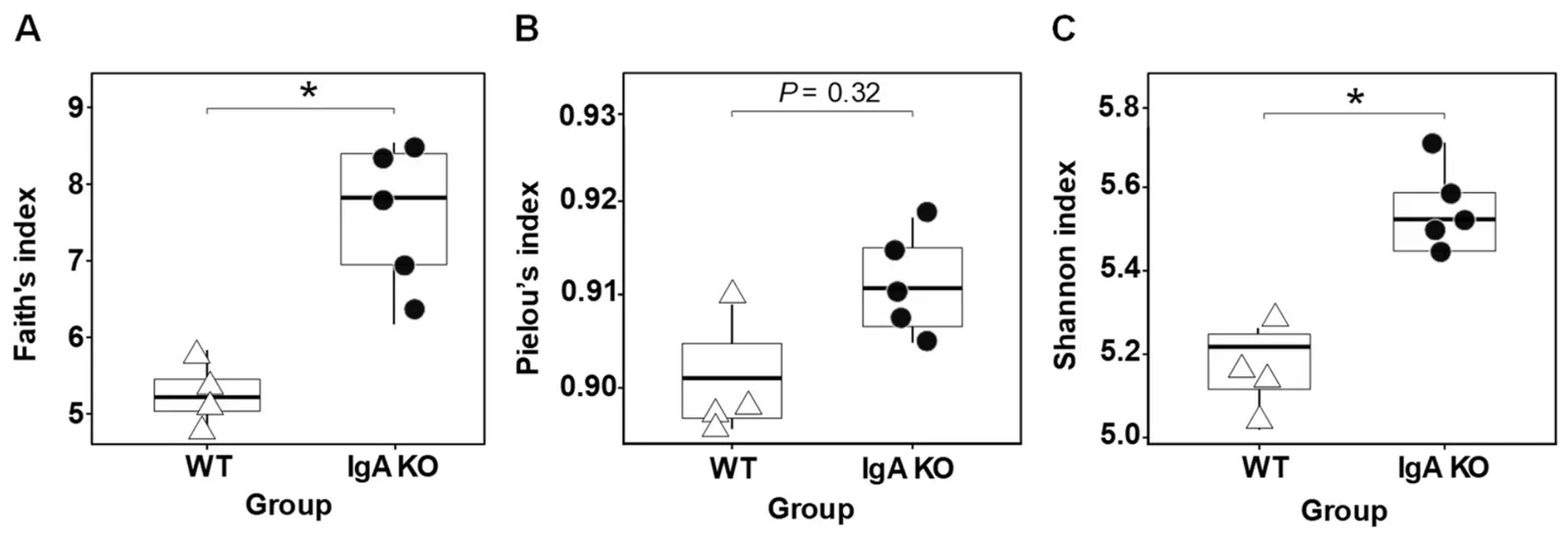
IgA deficiency altered alpha diversity of the gut microbiota. (**A**–**C**) Alpha diversity was compared between naïve WT (△) and IgA KO (●) mice by calculating the Faith’s phylogenetic diversity (PD) (bacterial richness), Pielou’s evenness (bacterial evenness), and Shannon (a combination of bacterial richness and evenness) indices. (**A**) Faith’s PD index. (**B**) Pielou’s evenness index. (**C**) Shannon index. The Faith’s PD indices were significantly higher in IgA KO mice than in WT mice. In the boxplots, the middle line, box, lower whisker, and upper whisker indicate the median, interquartile range, minimum, and maximum, respectively. * *p* < 0.05, Mann–Whitney *U* test. Each group was composed of four to five mice.

We examined how IgA deficiency could affect the gut bacterial compositions, using 16S rRNA amplicon sequencing of fecal samples from naïve WT and IgA KO mice, since IgA has been shown to play a central role in regulating the gut microbiota. We compared three alpha diversity indices between WT and IgA KO mice: the Faith’s PD index for the bacterial richness, the Pielou’s evenness index for the bacterial evenness, and the Shannon index for the combination of the bacterial richness and evenness. We found that alpha diversity measured by the Faith’s PD and Shannon indices was significantly higher in IgA KO mice (*p* < 0.01, Mann–Whitney *U* test, Figure 2A,C); the Pielou’s evenness indices were comparable between WT and IgA KO mice (Figure 2B).

**Figure 3:**
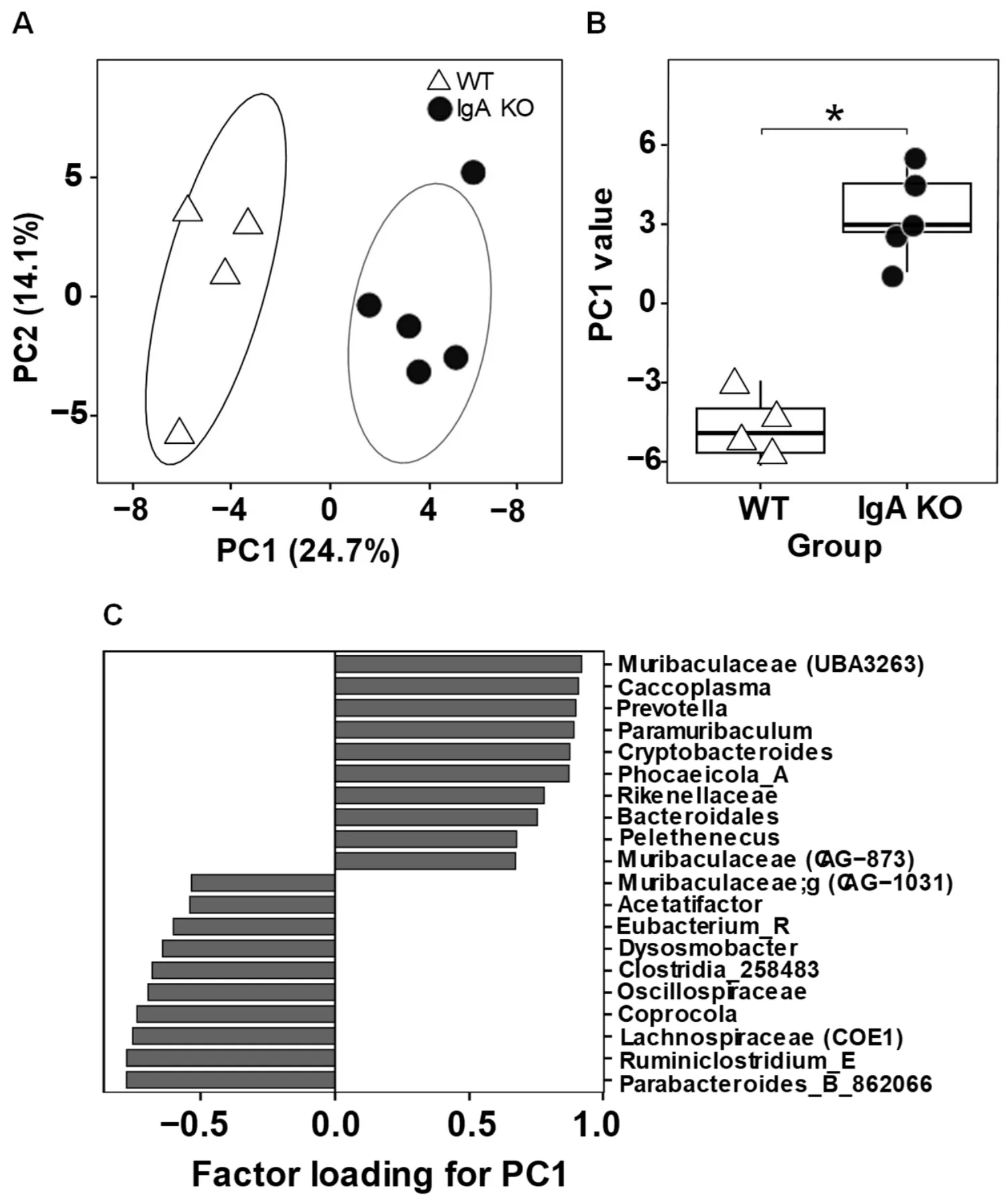
IgA deficiency altered the overall compositions of the gut microbiota at the genus level. (**A**–**C**) Principal component analysis (PCA) was performed using the relative abundance data from 16S rRNA amplicon sequencing at the genus level. (**A**) PCA clearly separated the microbiome profiles of naïve WT (△) and IgA KO (●) mice. The ellipses indicate the 80% confidence interval for each group. The values in parenthesis indicate the proportion of variance of each principal component (PC). (**B**) Distributions of IgA KO mice along the PC1 axis differed significantly from those of WT mice. In the boxplots, the middle line, box, lower whisker, and upper whisker represent the median, interquartile range, minimum, and maximum, respectively. * *p* < 0.05, Mann–Whitney *U* test. (**C**) Factor loadings for PC1 indicated that bacteria belonging to the order *Bacteroidales*, including *Muribaculaceae* (*UBA3263*), *Caccoplasma*, *Prevotella*, and *Paramuribaculum*, contributed to the separation along the PC1 axis. Each group was composed of four to five mice.

To determine the overall microbiome patterns among the fecal samples, we conducted PCA on the microbiome data and found a clear separation of microbiome profiles between the two groups on the principal component (PC) 1 axis at all taxonomic levels (*p* < 0.05, Mann–Whitney *U* test) (phylum, Supplementary Figure S1A; family, Supplementary Figure S1B; genus, Figure 3A,B; and species, Supplementary Figure S1C). At the genus level, the proportion of variance indicated that 24.7% and 14.1% of the variation in the microbiome dataset among the fecal samples could be attributed to the distributions on the PC1 and PC2 axes, respectively (Figure 3A). The factor loading of PC1 revealed that the genera belonging to the order *Bacteroidales*, including *Muribaculaceae* (*UBA3263*), *Caccoplasma*, and *Paramuribaculum* contributed to the separation on the PC1 axis (Figure 3C).

**Figure 4:**
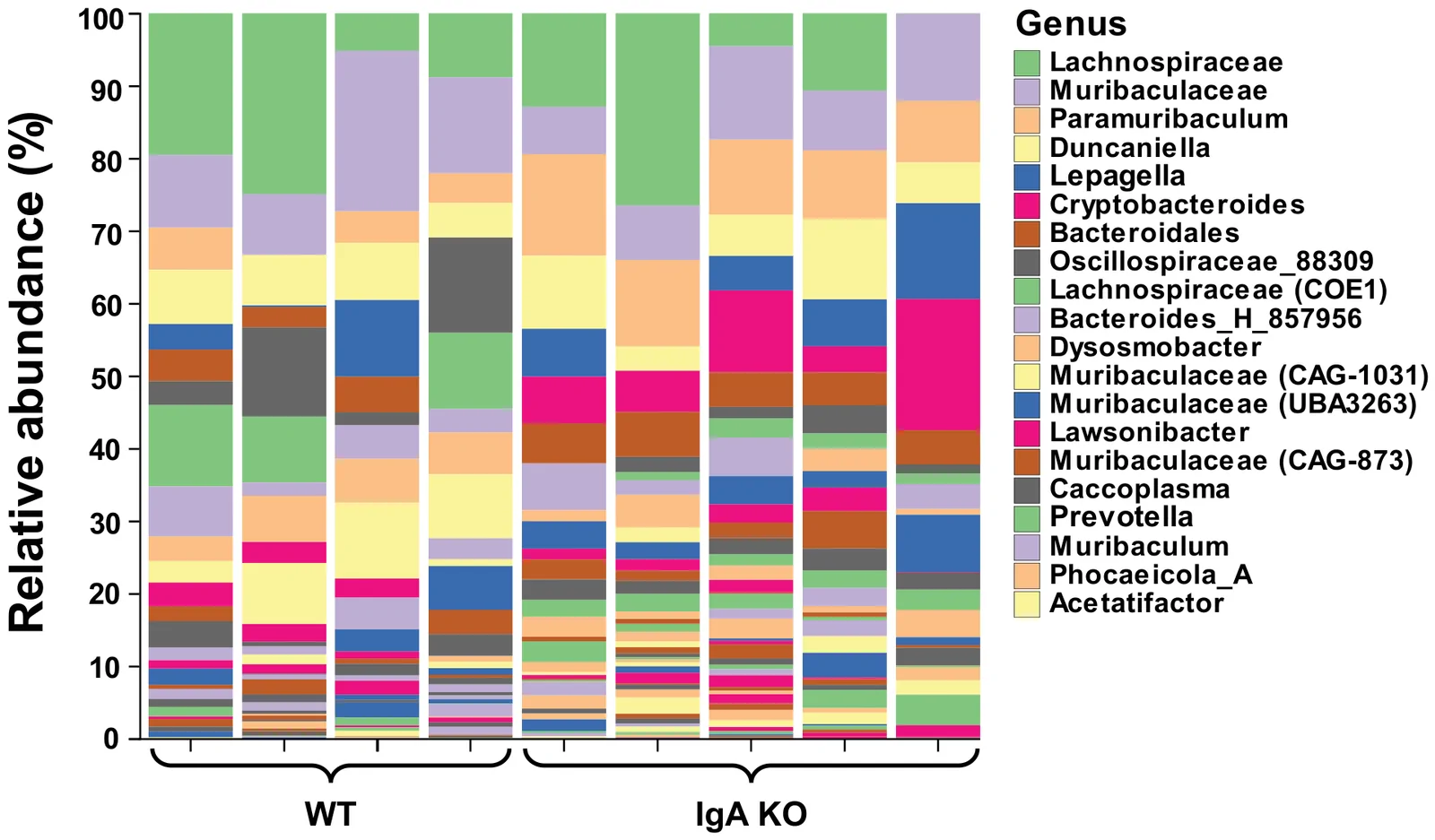
IgA deficiency altered the relative abundances of the gut microbiota at the genus level. Stacked bar plots show the relative abundances of bacterial taxa identified by 16S rRNA amplicon sequencing in WT and IgA KO mice. Each bar represents an individual mouse; colored segments represent the relative abundances of the indicated bacterial taxa. Each group was composed of four to five mice.

We also determined whether IgA deficiency could alter the relative abundances of bacterial taxa. The bacterial compositions were different between the two groups at all taxonomic levels (phylum, Supplementary Figure S2A and Supplementary Table S1; family, Supplementary Figure S2B and Supplementary Table S2; genus, Figure 4 and Table 2; and species, Supplementary Figure S2C and Supplementary Table S3). At the family level, compared with WT mice, the relative abundances of *Bacteroidales* (*UBA11471*) and *Bacteroidales* (*UBA932*), each of which was exclusively composed of the genera *Caccoplasma* and *Cryptobacteroides* (Table 2), respectively, were significantly higher in IgA KO mice than in WT mice (*p* < 0.05, Mann–Whitney *U* test, Supplementary Table S2). At the genus level, IgA KO mice had significantly higher relative abundances of bacteria belonging to the order *Bacteroidales*, including *Phocaeicola_A*, *Prevotella*, and the *Muribaculaceae* family bacteria [*Muribaculaceae* (*UBA 3263*), *Muribaculaceae* (*CAG-873*), and *Paramuribaculum*] than WT mice (*p* < 0.05, Mann–Whitney *U* test, Figure 4 and Table 2).

### 3.3. IgA Deficiency Exacerbates TMEV-IDD

**Figure 5:**
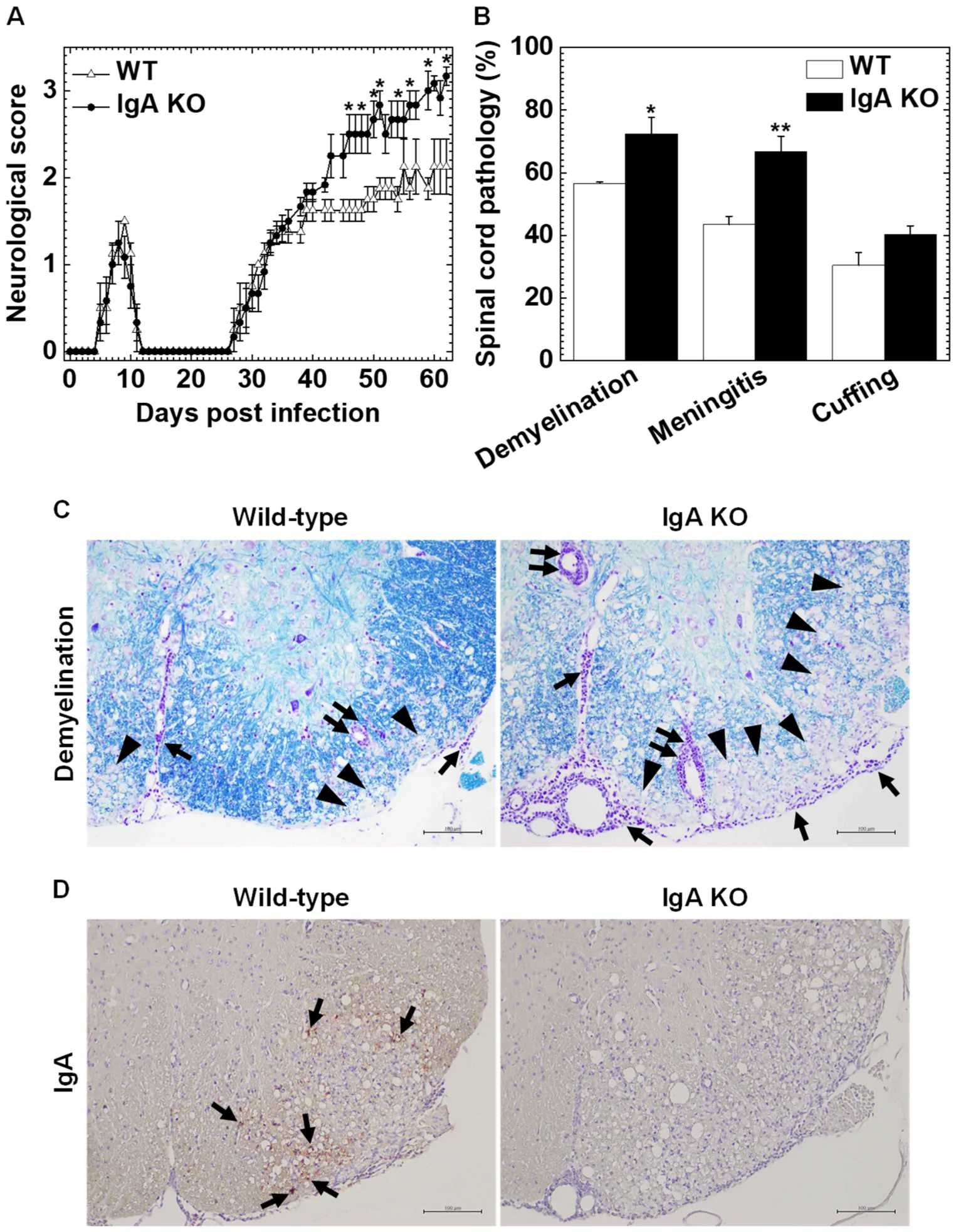
IgA deficiency exacerbated Theiler’s murine encephalomyelitis virus (TMEV)-induced demyelinating disease (TMEV-IDD). (**A**) Following TMEV infection, neurological signs, evaluated by impaired righting reflex scores, were monitored for 2 months. IgA KO mice (closed circle) exhibited significantly higher clinical scores during the chronic phase, 1 to 2 months post-infection (p.i.), but not during the acute phase, 1 week p.i., compared with WT mice (open triangle). Values are the mean ± SEM for four to six mice/group and are representative of two independent experiments. * *p* < 0.05, Mann–Whitney *U* test. (**B**) Quantification of spinal cord pathology scores. IgA KO mice (black bar) developed significantly more severe demyelination and meningitis in the spinal cord than WT mice (white bar) 2 months p.i. The severity of perivascular cuffing tended to be higher in IgA KO mice than in WT mice (*p* = 0.06). Values are the mean + SEM for four to six mice/group and are representative of two independent experiments. * *p* < 0.05 and ** *p* < 0.01, Student’s *t*-test. (**C**) Spinal cord sections were stained with Luxol fast blue to visualize myelin. Arrowheads, arrows, and paired arrows indicate demyelination, meningitis, and perivascular cuffing (inflammation), respectively. (**D**) IgA was visualized by immunohistochemistry with an anti-IgA antibody. IgA-positive (^+^) cell infiltration and IgA deposition in inflammatory demyelinating lesions were detected in WT mice, but not in IgA KO mice, 2 months p.i. Arrows indicate IgA staining. (**C**,**D**) Tissue sections are representative of two independent experiments comprising four to six mice/group. Scale bar = 100 μm.

TMEV-infected SJL/J mice are known to develop a biphasic disease [20]. During the acute phase, around 1 week p.i., infected mice develop polioencephalomyelitis primarily in the gray matter of the brain. During the chronic phase, 1 month p.i., the mice develop inflammatory demyelination in the white matter of the spinal cord, known as TMEV-IDD, a viral model of MS. To determine whether IgA deficiency could alter the TMEV-induced biphasic disease, we infected SJL/J WT and IgA KO mice i.c. with TMEV and monitored neurological signs by scoring impaired righting reflexes for 2 months. During the acute phase, both WT and IgA KO mice had similar impaired righting reflexes, reflecting polioencephalomyelitis, and recovered from the acute disease completely by 12 days p.i. (Figure 5A). During the chronic phase of TMEV infection, both WT and IgA KO mice exhibited progressive impairment of righting reflexes. Although there was no difference in the onset of TMEV-IDD between the two groups (mean onset days ± SEM: WT, 29.0 ± 0.9; IgA KO, 29.5 ± 0.9), IgA KO mice had significantly higher righting reflex scores than WT mice (*p* < 0.05, Mann–Whitney *U* test, Figure 5A).

To investigate whether IgA deficiency could alter pathological changes in the CNS during the chronic phase, 2 months p.i., we stained CNS sections with Luxol fast blue and compared pathological scores between WT and IgA KO mice. In the spinal cord, we observed demyelination, meningitis, and perivascular cuffing (inflammation), mainly in the ventral and lateral funiculi, in both groups (Figure 5C). Consistent with the impaired righting reflex scores, IgA KO mice had significantly higher pathology scores for demyelination and meningitis than WT mice (*p* < 0.05, Student’s *t*-test, Figure 5B). The level of perivascular cuffing was also higher in IgA KO mice than in WT mice, although it did not reach a significant difference (*p* = 0.06). In the brain, the pathology scores were higher in IgA KO mice than in WT mice, although it did not reach statistical significance: mean brain pathology scores ± SEM: WT, 3.3 ± 0.3; IgA KO, 3.8 ± 0.5. We also conducted immunohistochemistry against IgA and confirmed that WT mice had IgA^+^ cells and IgA deposition in inflammatory demyelinating lesions; as expected, IgA KO mice had no IgA staining (Figure 5D).

### 3.4. IgA Deficiency Enhances CNS Viral Persistence and T Cell Infiltration in TMEV-IDD

**Figure 6:**
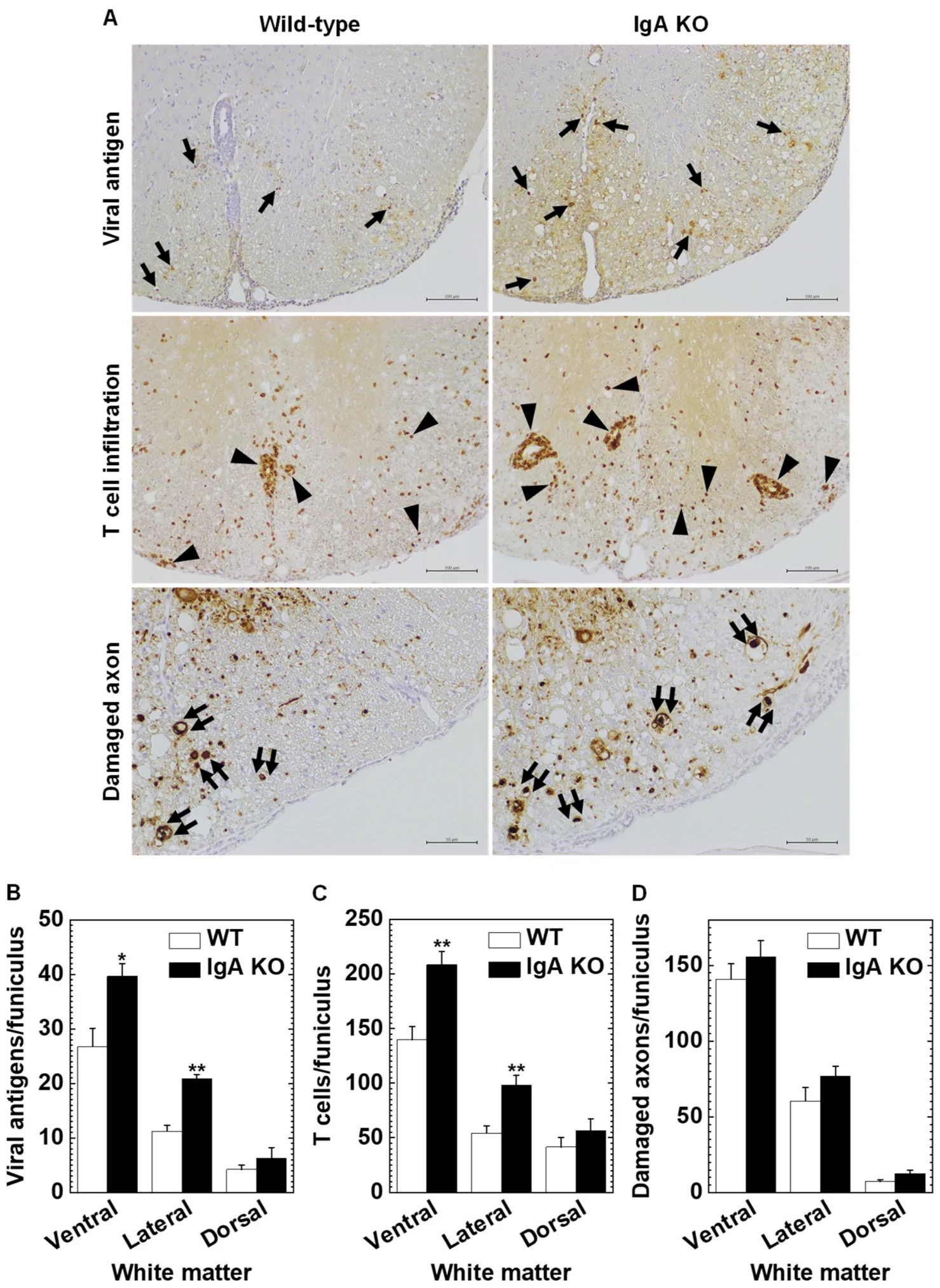
IgA deficiency increased viral persistence and T cell infiltration in the spinal cord, but not axonal damage, in TMEV-IDD. (**A**) Viral antigens, T cell infiltration, and damaged axons in the spinal cord were visualized by immunohistochemistry with antibodies against TMEV (top panels), CD3 (middle panels), and non-phosphorylated neurofilaments (bottom panels), respectively. In both WT (left panels) and IgA KO (right panels) mice, TMEV antigen^+^ cells, CD3^+^ T cells, and damaged axons were mainly localized in the white matter of the spinal cord 2 months p.i. Arrows, arrowheads, and paired arrows indicate TMEV antigen^+^ cells, T cells, and damaged axons, respectively. Tissue sections are representative of two independent experiments comprising four to six mice/group. Scale bar = 100 μm (viral antigen and T cell infiltration) and 50 μm (damaged axon). (**B**–**D**) Quantification of viral antigen^+^ cells, T cells, and damaged axons in the spinal cord. IgA KO mice (black bar) had significantly higher levels of viral antigen^+^ cells and T cell infiltration, but not damaged axons, in the spinal cord compared with WT mice (white bar) 2 months p.i. Values are the mean + SEM of four to six mice/group and are representative of two independent experiments. * *p* < 0.05 and ** *p* < 0.01, Student’s *t*-test.

Since viral persistence plays a pathogenic role in TMEV-IDD [43], we visualized TMEV antigen^+^ cells by immunohistochemistry using an anti-TMEV antibody and compared the number of viral antigen^+^ cells in the spinal cord between the two groups. TMEV antigen^+^ cells were primarily localized in the white matter of the ventral and lateral funiculi of the spinal cord (Figure 6A, top panels). IgA KO mice had significantly more viral antigen^+^ cells in the white matter of the ventral and lateral funiculi than WT mice (ventral, *p* < 0.05; lateral, *p* < 0.01; Student’s *t*-test, Figure 6B). In the white matter of the dorsal funiculus, the number of viral antigen^+^ cells was also higher in IgA KO mice than in WT mice, although it did not reach statistical difference.

We also visualized T cells by immunohistochemistry using an anti-CD3 antibody and found that T cells were primarily detected in the white matter and meninges of the spinal cord in both groups (Figure 6A, middle panels). We found significantly greater T cell infiltration in the ventral and lateral funiculi, but not the dorsal funiculus, of the spinal cord in IgA KO mice than in WT mice (Figure 6C). To further determine whether IgA^+^ cells could colocalize with T cells in CNS lesions, we performed double immunofluorescent staining with anti-IgA and anti-CD3 antibodies. We found that most IgA^+^ cells were observed sporadically in demyelinating lesions and did not colocalize with T cells. In the meningeal and perivascular spaces, a small number of IgA^+^ cells were observed adjacent to T cells (Supplementary Figure S3).

Axonal degeneration in the spinal cord has also been associated with neurological scores in TMEV-IDD [44]. We visualized damaged axons in the spinal cord using immunohistochemistry for non-phosphorylated neurofilaments. In both groups, axonal degeneration was mainly observed in the ventral and lateral funiculi of the spinal cord (Figure 6A, bottom panels). Although we observed more damaged axons in IgA KO mice than in WT mice, there were no significant differences between the two groups (Figure 6D).

### 3.5. IgA Deficiency Alters Cytokine Profiles but Not Anti-Viral Immune Responses

**Figure 7:**
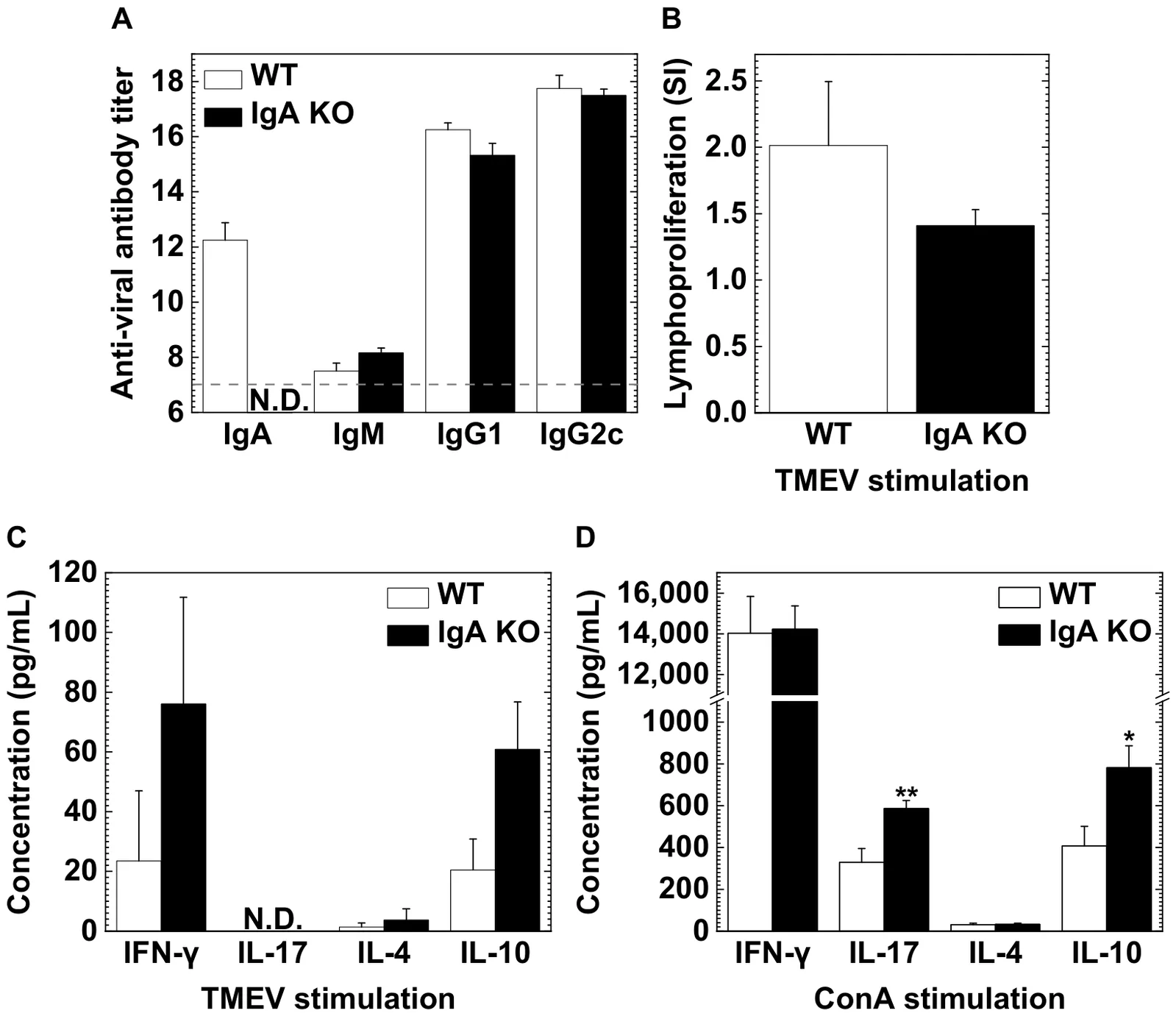
IgA deficiency enhanced the production of interleukin (IL)-17 and IL-10, but not anti-TMEV lymphoproliferative or antibody isotype responses, in TMEV-IDD. (**A**) Levels of serum anti-TMEV antibody isotypes were determined by ELISAs. Anti-TMEV IgM, IgG1, and IgG2c titers were comparable between WT (white bar) and IgA KO (black bar) mice 2 months p.i.; anti-TMEV IgA responses were mounted in WT mice, but not in IgA KO mice. Values are the mean + SEM of four to six mice/group and are representative of two independent experiments. The dotted line indicates the detection limit. N.D., not detectable. (**B**) Lymphoproliferative responses to TMEV were assessed using the Cell Counting Kit-8 and expressed as the stimulation index (SI): (mean absorbance in TMEV stimulation)/(mean absorbance without stimulation). The levels of TMEV-specific lymphoproliferation were comparable between WT and IgA KO mice 2 months p.i. Values are the mean + SEM of three pools of the spleens from two mice (six mice/group) and are representative of two independent experiments. (**C**,**D**) Concentrations of interferon (IFN)-γ, IL-17, IL-4, and IL-10 were quantified by ELISAs. (**C**) Under TMEV stimulation, concentrations of IFN-γ and IL-10 were higher in IgA KO mice than in WT mice, although there were no statistical differences between WT and IgA KO mice. (**D**) Under mitogen concanavalin A (ConA) stimulation, IgA KO mice had significantly higher concentrations of IL-17 and IL-10 than WT mice. Values are the mean + SEM of five to six pools of the spleen from two mice (10 to 12 mice/group). * *p* < 0.05 and ** *p* < 0.01, Student’s *t*-test.

Anti-viral immune responses can play both protective and pathogenic roles in TMEV infection [45]. We examined whether IgA deficiency could alter anti-viral humoral and cellular responses. We compared serum anti-TMEV antibody isotype titers between the two groups using ELISAs. Anti-TMEV IgM, IgG1, and IgG2c titers were comparable between the two groups. As expected, we detected substantial serum anti-TMEV IgA titers in WT mice, but not in IgA KO mice (Figure 7A). To determine whether IgA deficiency could affect antigen-specific T cell responses, splenic MNCs were isolated from TMEV-infected WT and IgA KO mice and stimulated with TMEV [46]. Using a CCK-8 assay, we compared TMEV-specific lymphoproliferative responses, which reflect anti-TMEV T cell responses, between WT and IgA KO mice. Both groups exhibited substantial anti-TMEV lymphoproliferation, with no statistically significant difference between them (Figure 7B).

We also compared the concentrations of four Th-related cytokines between WT and IgA KO mice using ELISAs. Following TMEV stimulation, splenic MNCs from IgA KO mice produced higher levels of IFN-γ and IL-10 than those from WT mice, although the differences did not reach statistical significance (Figure 7C). IL-17 production was not detectable in either group; IL-4 production was comparable between the two groups. Under ConA stimulation, IgA KO mice had significantly higher levels of IL-17 and IL-10 than WT mice (Figure 7D). In contrast, IFN-γ and IL-4 levels were comparable between the two groups.

## 4. Discussion

IgA plays a central role in regulating the composition of the gut microbiota, whose dysbiosis has been associated with a variety of health and disease conditions. Selective IgA deficiency (sIgAD) is considered the most common inborn error of immunity and is more common in Caucasians. The prevalence of sIgAD varies by ethnic background; for example, 1:163 in Spain and 1:18,550 in Japan [47,48]. Despite the high prevalence of sIgAD in the world, there have been two case reports that patients with sIgAD developed MS [47,49,50]. To clarify the role of IgA in MS, we generated IgA KO mice on the background of SJL/J mice, which have been used to induce several MS models, including the TMEV model and autoimmune EAE models induced with myelin proteolipid protein (PLP) and myelin oligodendrocyte glycoprotein (MOG). We found that serum IgM and IgG1 concentrations were comparable between naïve WT and IgA KO mice; serum IgG2c levels were significantly higher in naïve IgA KO mice than in naïve WT mice (Figure 1A). Although IgA KO mice at 4 to 5 weeks of age weighed less than WT mice, IgA KO mice caught up to the weight of WT mice during the 2-month observation period. Histologically, we found no abnormalities in general organs, including the thymus, spleen, and intestine (Figure 1B).

In humans, Moll et al. demonstrated that sIgAD individuals exhibited significantly decreased alpha diversity of the gut microbiome compared with matched-control individuals; overall microbiome patterns differed significantly between sIgAD and matched-control IgA^+^ household individuals [51]. On the other hand, Catanzaro et al. reported no significant difference in alpha diversity of the fecal microbiome between sIgAD individuals and healthy controls, although sIgAD individuals had altered gut microbiota compositions compared with healthy controls [52]. Conrey et al. found comparable alpha diversity and overall microbiome profiles between sIgAD individuals and IgA^+^ co-housed siblings [53]. In the current study, we analyzed the gut microbiota and found that IgA KO mice had increased alpha diversity compared with WT mice (Figure 2). Although our data were consistent with the clinical findings reported by Moll et al., how IgA deficiency could influence alpha diversity of the gut microbiota remains controversial.

PCA of the microbiome data clearly separated the IgA KO mouse group from the WT mouse group; bacterial genera, including *Cryptobacteroides*, *Caccoplasma*, *Prevotella*, *Phocaeicola*, and the *Muribaculaceae* family (formerly *S24-7*) bacteria [(*Muribaculaceae* (*UBA 3263*), *Muribaculaceae* (*CAG-873*), and *Paramuribaculum*], most of which belong to the order *Bacteroidales*, contributed to the separation (Figure 3) [54,55,56]. Most of these bacterial taxa exhibited significant changes in the relative abundances of IgA KO mice (Figure 4, Table 2, and Supplementary Tables S1–S3). These bacterial taxa can modulate the production of short-chain fatty acids, which regulate immune responses [57,58,59]. The bacterial taxa changed in IgA KO mice in the SJL/J background were different from those reported in IgA KO mice in the C57BL/6 background, except for *Prevotella* [31]. This could be due to differences in the microbiota compositions or in the natural IgA antibody repertoire between SJL/J and C57BL/6 mice. Since gut microbiota compositions have been shown to modulate immune responses, including Th17 responses [60], the altered microbiota compositions in IgA KO mice could contribute to several aspects of immune responses examined in the current study during TMEV infection.

TMEV infection in SJL/J mice induces a biphasic disease [61]: during the acute phase, TMEV predominantly infects neurons in the brain, causing polioencephalomyelitis; during the chronic phase, TMEV preferentially infects glial cells and macrophages in the spinal cord, causing TMEV-IDD. In the current study, although both WT and IgA KO mice developed a similar biphasic disease course following TMEV infection, IgA deficiency exacerbated neurological signs during the chronic phase, but not during the acute phase (Figure 5A). Thus, IgA seemed to play a role only during the chronic phase of TMEV infection. This can be explained by the different levels of IgA in the CNS during the time course of TMEV infection. In TMEV infection, we previously demonstrated that IgA deposition and IgA^+^ cells were observed in the CNS only during the chronic phase and that CNS *Igha* gene expression was significantly higher during the chronic phase than during the acute phase [25].

Histologically, we demonstrated that IgA deficiency led to more severe inflammatory demyelination, with increased TMEV antigen^+^ cells and T cells in the spinal cord of TMEV-infected mice (Figure 5B,C and Figure 6A–C). These findings were consistent with previous reports that both viral persistence and T cell infiltration in the spinal cord could contribute to the development of TMEV-IDD [21,62]. On the other hand, IgA KO mice had only slightly increased axonal damage in the spinal cord (Figure 6A,D). In TMEV infection, axonal degeneration has been shown to precede inflammatory demyelination (the Inside-Out model), where damaged axons were detectable in the white matter of the spinal cord during the acute phase, as early as 1 week p.i. [63]. In the Inside-Out model, early neuronal TMEV infection causes Wallerian degeneration of axons, leading to late demyelination along the degenerated neural tract. Since the severity of acute neuronal disease in IgA KO mice was similar to that in WT mice, comparable neuronal damage during the acute phase may have led to a similar level of axonal damage during the chronic phase of TMEV infection.

In TMEV infection, anti-viral antibodies have been shown to contribute to viral clearance from the CNS. Following intracerebral TMEV inoculation, administration of an anti-TMEV monoclonal antibody to athymic nude mice, which cannot mount T cell or antibody responses, reduced CNS viral titers and protected against lethal infection [18]. Similarly, in Sindbis virus infection in severe combined immunodeficient (SCID) mice, which have neither functional T cells nor antibodies, passive transfer of antibodies against the virus resulted in viral clearance from the CNS, although adoptive transfer of anti-viral T cells had no effect on viral clearance [64]. In the current study, only TMEV-infected WT mice mounted substantial IgA responses to the virus, although IgA KO mice had comparable levels of anti-TMEV lymphoproliferative responses and serum anti-TMEV IgM, IgG1, and IgG2c titers compared with WT mice (Figure 7A,B). Thus, the greater number of TMEV antigen^+^ cells in the spinal cord of IgA KO mice than in WT mice (Figure 6A,B) could reflect suppression of CNS viral spread by anti-TMEV IgA in WT mice. These results also suggest that although naïve IgA KO mice had significantly higher serum whole IgG2c levels than naïve WT mice, IgA deficiency would not alter the antigen-specific IgM, IgG1, or IgG2c responses following TMEV infection.

Regarding cytokine responses, we found that IgA KO mice exhibited higher levels of pro-inflammatory IFN-γ and IL-17 in response to TMEV and ConA stimulation, respectively, than WT mice (Figure 7C,D). Thus, IgA deficiency may exacerbate TMEV-IDD by enhancing IFN-γ and IL-17 production. In TMEV-IDD, IFN-γ has been reported to play both protective and pathogenic roles. Blockade of IFN-γ with a neutralizing antibody against IFN-γ, either before or after TMEV infection, exacerbated inflammatory demyelination and increased viral persistence in the spinal cord [65]. In contrast, administration of recombinant IFN-γ during the chronic phase accelerated TMEV-IDD onset by enhancing anti-TMEV Th1 responses [66]. Anti-IL-12 neutralizing antibody treatment reduced IFN-γ production by splenic cells from TMEV-infected mice, resulting in decreased severity of TMEV-IDD, both clinically and neuropathologically [67]. On the other hand, IL-17 has been shown to play only a pathogenic role in TMEV-IDD. Injection of an anti-IL-17 neutralizing antibody in SJL/J mice reduced the incidence of TMEV-IDD with decreased levels of infectious virus and IL-17-producing-cell infiltration in the CNS [22]. Although C57BL/6 WT mice are resistant to TMEV-IDD, C57BL/6 RORγt (retinoic-acid-receptor-related orphan receptor-γt) transgenic mice, which have a Th17-biased response, developed inflammatory demyelination in the spinal cord similar to that observed in SJL/J mice [21].

We also found that TMEV-infected IgA KO mice had higher levels of IL-10 production in both TMEV and ConA stimulation than TMEV-infected WT mice (Figure 7C,D). IL-10 has been shown to play either a pathogenic or protective role in TMEV infection, depending on the time course. We previously demonstrated that, during the acute phase, ex vivo-induced regulatory T cell (iTreg) injection enhanced IL-10 production in the periphery; iTreg injection exacerbated clinical scores in acute polioencephalomyelitis and increased viral replication in the brain [68]. During the chronic phase, iTreg injection enhanced IL-10 production from splenic MNCs in TMEV stimulation, resulting in less severe demyelinating lesions and T cell infiltration without changes in viral persistence in the spinal cord. Uhde et al. demonstrated that administration of an anti-IL-10 receptor (IL-10R) antibody on days 0, 7, 14, and 21 after TMEV infection accelerated the onset of TMEV-IDD compared with administration of the control antibody, although the authors did not find any histological differences between the control and anti-IL-10R antibody groups [69]. On the other hand, anti-IL-10R treatment 35 and 42 days after TMEV infection exacerbated clinical scores and increased T cell infiltration in the spinal cord, compared with the control treatment. Although we did not quantify the concentrations of IL-10 during the acute phase, the current study suggests that increased IL-10 production in IgA KO mice may be associated with increased CNS viral persistence, exacerbating TMEV-IDD.

The current study had two limitations. First, although we analyzed the gut microbiota in fecal samples from naïve IgA KO mice, we did not analyze fecal samples after TMEV infection. We previously reported that TMEV infection altered the gut microbiota and that the altered bacterial composition was associated with *Igha* gene expression in the CNS [25]. Thus, it would be intriguing to determine which CNS gene expression correlates with the gut bacterial composition under IgA deficiency, using fecal samples from TMEV-infected IgA KO mice. This will further clarify the impact of IgA deficiency on the gut microbiota in TMEV-IDD.

Second, we did not clarify the precise role of IgA^+^ B cells in TMEV-IDD. In MS and EAE, IgA^+^ B cells were reported to have a suppressive effect on autoreactive T cells by producing IL-10 [32,33]. In the current study, however, we found no difference in lymphoproliferative responses to TMEV between WT and IgA KO mice using MNCs. Since the MNCs from WT mice contain both IgA^+^ B cells and TMEV-specific T cells, this result suggests that IgA^+^ B cells did not affect TMEV-specific T cell proliferation. Nevertheless, to clarify whether IL-10-producing IgA^+^ B cells, as reported in MS and EAE, could play a role in TMEV infection, experiments are needed, such as co-culturing T cells either with IgA^+^ or IgA^−^ B cells in the presence or absence of an anti-IL-10 antibody.

In conclusion, we generated IgA KO mice on the SJL/J background and demonstrated changes in the gut microbiota compositions with increases in alpha diversity. Although decreased alpha diversity has been reported to contribute to the pathogenesis of microbiota-mediated diseases, including IBD and obesity [70,71], changes in alpha diversity in MS were inconsistent. Miyake et al. reported no significant changes in alpha diversity between MS patients and healthy individuals [72]; Kaur et al. showed decreased alpha diversity in MS patients compared with healthy controls [73]. To clarify the role of IgA in MS, we infected IgA KO mice with TMEV, which resulted in more severe TMEV-IDD than WT mice clinically and neuropathologically, with increased viral persistence and IL-17/IL-10 production. Therefore, in the TMEV model, IgA may play a protective role by neutralizing TMEV in the CNS of WT mice. Alternatively, although the causal relationship between alterations in the gut microbiota and exacerbation of TMEV-IDD remains unclear, IgA deficiency-mediated gut dysbiosis may contribute to enhanced immunopathology in IgA KO mice by dysregulating immune balance.

## Figures and Tables

**Table 1:** The primer sets for two-step tailed polymerase chain reaction.

Primer Set		Sequence (5′ → 3′)
First primer	Forward (1st-341f)	ACACTCTTTCCCTACACGACGCTCTTCCGATCT-NNNNN-CCTACGGGNGGCWGCAG
	Reverse (1st-805r)	GTGACTGGAGTTCAGACGTGTGCTCTTCCGATCT-NNNNN-GACTACHVGGGTATCTAATCC
Second primer	Forward (2ndF)	AATGATACGGCGACCACCGAGATCTACAC-Index*-ACACTCTTTCCCTACACGACGC
	Reverse (2ndR)	CAAGCAGAAGACGGCATACGAGAT-Index*-GTGACTGGAGTTCAGACGTGTG

Index*, sequencing for sample identification.

**Table 2:** Differentially abundant bacterial genera between wild-type (WT) and IgA knockout (KO) mice.

Genus	WT Mice (%) *	IgA KO Mice (%)	*p* Value ^#^
*Paramuribaculum*	3.57 ± 2.49	10.86 ± 2.17	<0.05
*Cryptobacteroides*	0	8.99 ± 5.81	<0.05
*Muribaculaceae* (*UBA3263*)	0	4.02 ± 2.30	<0.05
*Caccoplasma*	0	2.46 ± 0.48	<0.05
*Prevotella*	0	2.31 ± 0.45	<0.05
*Muribaculaceae* (*CAG-873*)	0	2.27 ± 1.90	<0.05
*Phocaeicola_A*	0	2.06 ± 1.22	<0.05
*Muribaculaceae* (*UBA7173*)	0	0.93 ± 0.83	<0.05
*Helicobacter_C*	0	0.80 ± 0.97	<0.05
*Dysosmobacter*	5.39 ± 1.36	1.98 ± 1.81	<0.05
*Parabacteroides_B_862066*	2.21 ± 1.36	0	<0.05
*Ruminiclostridium_E*	0.78 ± 0.64	0	<0.05
*Parasutterella_564865*	0.62 ± 0.67	0	<0.05
*Coprocola*	0.37 ± 0.32	0	<0.05

* Values are presented as the mean ± standard deviation (SD) of the relative abundances (%). Complete bacterial lists were shown in the Table S4.csv file as a Supplementary Material. Each group was composed of four to five mice. ^#^ The relative abundances were compared using the Mann–Whitney *U* test.

## Data Availability

The original data presented in the current study are openly available in the SRA at https://www.ncbi.nlm.nih.gov/bioproject/PRJNA1510292/ (accessed on 10 August 2026).

## Supplementary Materials

The following supporting information can be downloaded at: https://www.mdpi.com/article/10.3390/cells15181665/s1, Text S1: Donor DNA vector construction.; Figure S1: PCA of the gut microbiota in naïve SJL/J WT and IgA KO mice at different taxonomic levels; Figure S2: Relative abundances of bacteria in the fecal samples from naïve SJL/J WT and IgA KO mice at the phylum, family, and species levels; Figure S3: Double immunofluorescent staining against IgA+ and CD3+ T cells in the spinal cord section of SJL/J WT and IgA KO 2-months, after infection; Table S1: Lists of significantly increased or decreased bacteria in the fecal samples from IgA KO mice at the phylum level; Table S2: Lists of significantly increased or decreased bacteria in the fecal samples from IgA KO mice at the family level; Table S3: Lists of significantly increased or decreased bacteria in the fecal samples from IgA KO mice at the species level; Table S4 file: Complete bacterial lists in the fecal samples from WT and IgA KO mice at the genus level; Table S1 file: Complete bacterial lists in the fecal samples from WT and IgA KO mice at the phylum level; Table S2 file: Complete bacterial lists in the fecal samples from WT and IgA KO mice at the family level; Table S3 file: Complete bacterial lists in the fecal samples from WT and IgA KO mice at the species level.
