# Supplementary material for: IgA Deficiency Exacerbates an Animal Model of Multiple Sclerosis Induced with Theiler’s Virus
Source: Cells. 2026 Sep 15;15(18):1665. doi: 10.3390/cells15181665 (PMC13605160; doi:10.3390/cells15181665)
Supplement: Supplementary file 1 [file cells-15-01665-s001.zip › Supplementary Figures and Tables.pdf]

Supplementary Materials

Table of contents

Supplementary Figure S1. Principal component analysis (PCA) of the gut microbiota in naïve SJL/J wild-type (WT) and IgA knockout (KO) mice at different taxonomic levels …2

Supplementary Figure S2. Relative abundances of bacteria in the fecal samples from naïve SJL/J WT and IgA KO mice at the phylum, family, and species levels …3

Supplementary Figure S3. Double immunofluorescent staining against IgA^+ and $CD3^+$ T cells in the spinal cord section of SJL/J WT and IgA KO 2-months, after infection …4

Supplementary Table S1. Lists of significantly increased or decreased bacteria in the fecal samples from IgA KO mice at the phylum level …5

Supplementary Table S2. Lists of significantly increased or decreased bacteria in the fecal samples from IgA KO mice at the family level …6

Supplementary Table S3. Lists of significantly increased or decreased bacteria in the fecal samples from IgA KO mice at the species level …7

Supplementary Table S1 file. Complete bacterial lists in the fecal samples from WT and IgA KO mice at the phylum level …CSV file

Supplementary Table S2 file. Complete bacterial lists in the fecal samples from WT and IgA KO mice at the family level …CSV file

Supplementary Table S3 file. Complete bacterial lists in the fecal samples from WT and IgA KO mice at the species level …CSV file

Supplementary Table S4 file. Complete bacterial lists in the fecal samples from WT and IgA KO mice at the genus level …CSV file

Supplementary Text S1. Donor DNA vector construction …PDF file

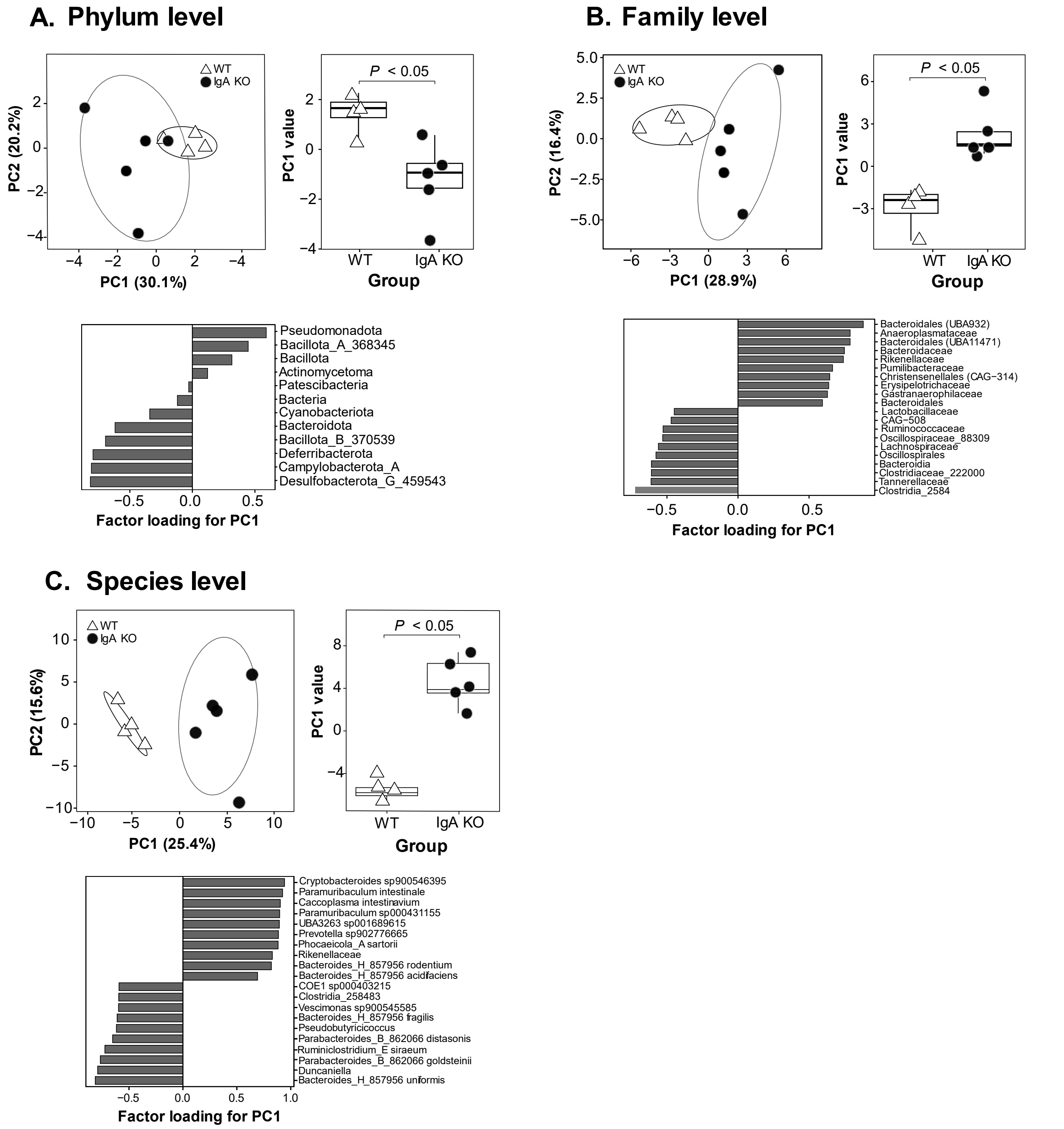

Supplementary Figure S1. Principal component analysis (PCA) of the gut microbiota in naïve SJL/J wild-type (WT) and IgA knockout (KO) mice at different taxonomic levels. (**A–C**) PCA was performed using the relative abundance data from 16S rRNA amplicon sequencing at the phylum (**A**), family (**B**), and species (**C**) levels. Upper left panel: PCA clearly separated the microbiome profiles of WT (△) and IgA KO (●) mice at each taxonomic level. The ellipses indicate the 80% confidence interval for each group. The values in parenthesis indicate the proportion of variance of each principal component (PC). Upper right panel: distributions of IgA KO mice along the PC 1 axis differed significantly from those of WT mice. In the boxplots, the middle line, box, lower whisker, and upper whisker represent the median, interquartile range, minimum, and maximum, respectively. The statistical analysis of PC1 values was performed using the Mann–Whitney *U* test. Lower panel: factor loading for PC1 indicates the bacterial taxa contributing positively or negatively to PC1. Each group was composed of four to five mice.

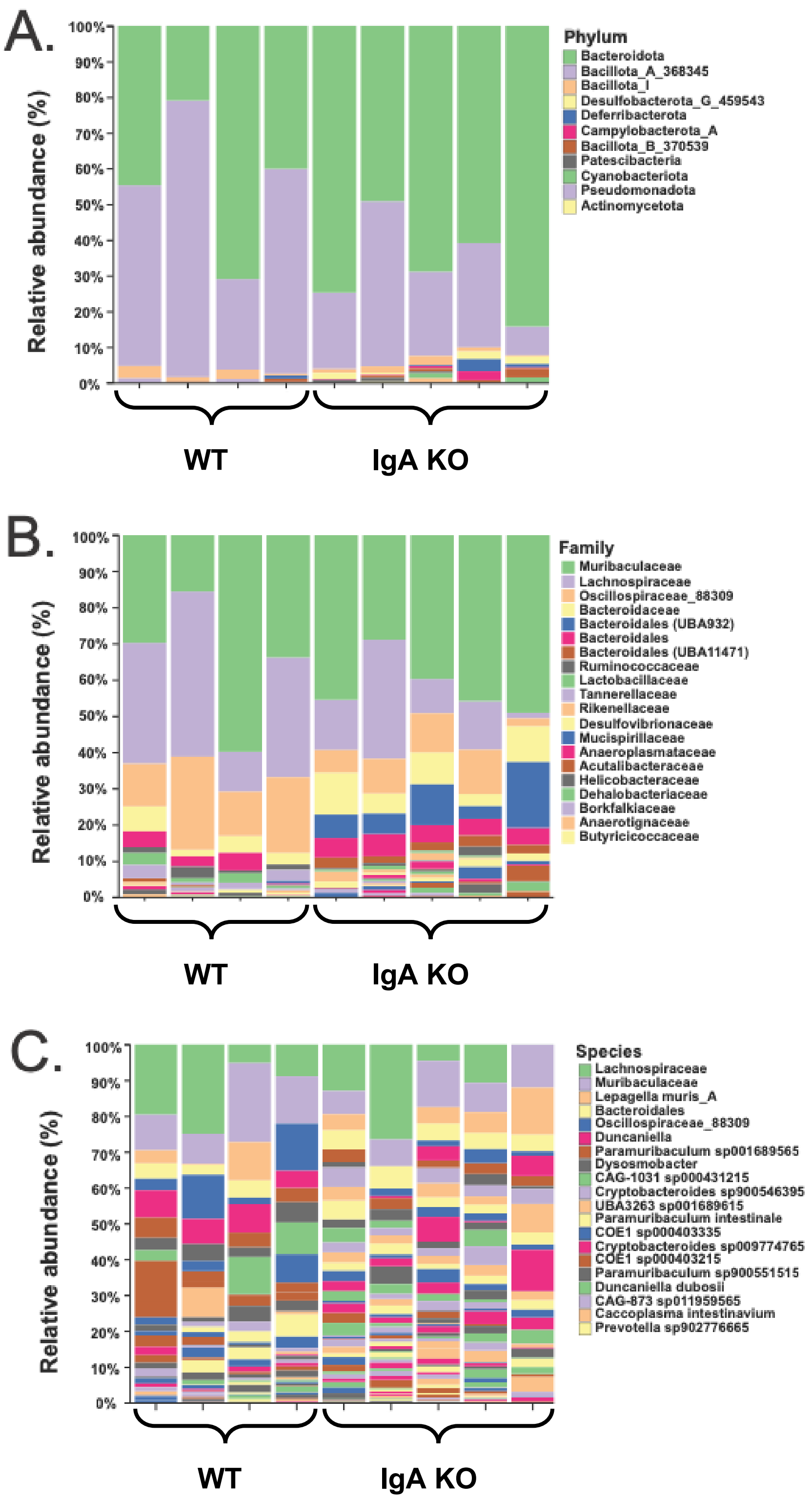

Supplementary Figure S2. Relative abundances of bacteria in the fecal samples from naïve SJL/J WT and IgA KO mice at the phylum, family, and species levels. (**A–C**) Stacked bar plots show the relative abundances of bacterial taxa identified by 16S rRNA amplicon sequencing in WT and IgA KO mice at the phylum (**A**), family (**B**), and species (**C**) levels. Each bar represents an individual mouse; colored segments represent the relative abundances of the indicated bacterial taxa. Each group was composed of four to five mice.

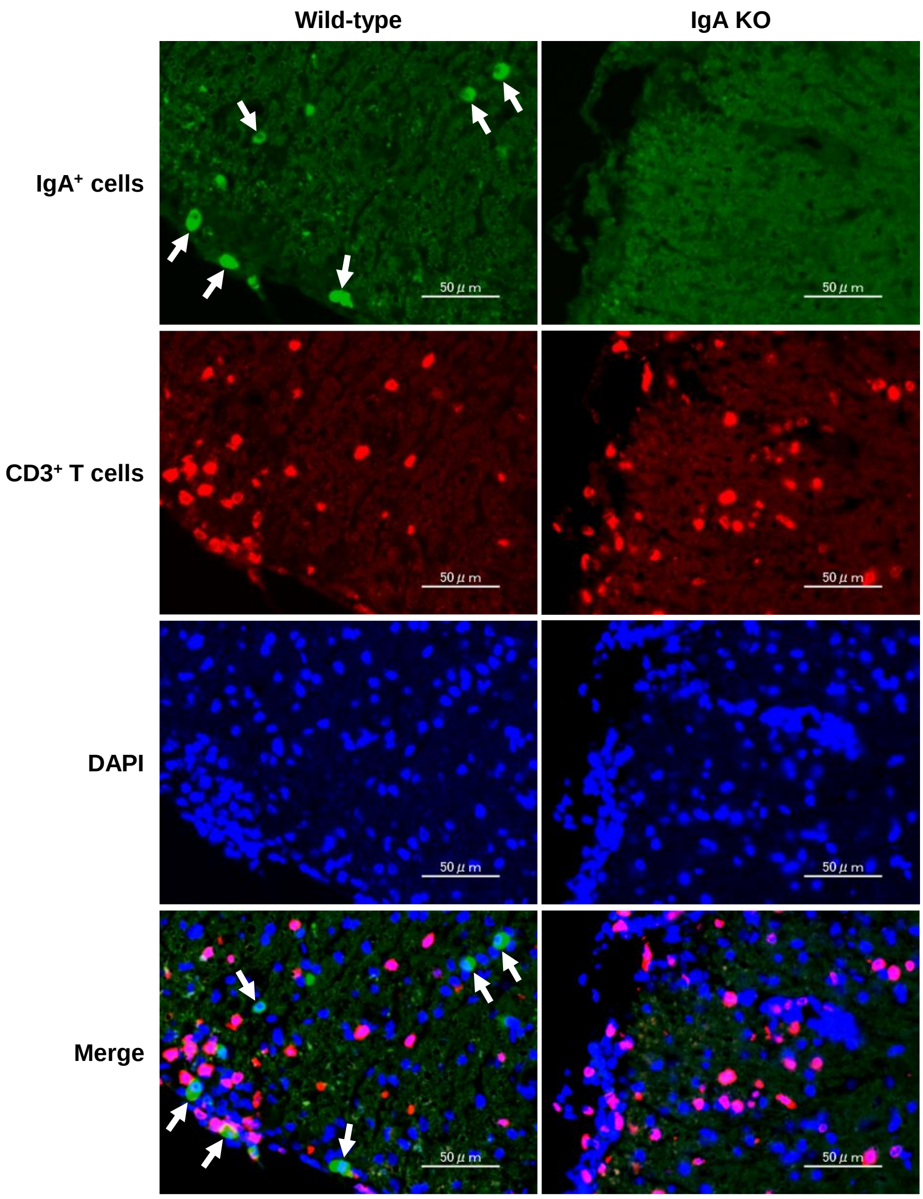

Supplementary Figure S3. Double immunofluorescent staining for IgA^{+} and CD3^{+} T cells in spinal cord sections from SJL/J WT and IgA KO mice 2 months after infection. SJL/J WT and IgA KO mice were infected intracerebrally with Theiler's murine encephalomyelitis virus (TMEV). Two months after infection, mice were killed with isoflurane and perfused with phosphate-buffered saline (PBS), followed by perfusion with 4% paraformaldehyde in PBS. For histological analysis, the spinal cord was harvested and embedded in paraffin. We stained 4-μm-thick tissue sections with anti-IgA and anti-CD3 antibodies and labeled with Alexa Fluor 488 (green) and Alexa Fluor 555 (red), respectively. DAPI (4́,6-diamidino-2-phenylindole) was used to visualize nuclei (blue). Arrows indicate IgA^{+} cells (top and bottom panels). Tissue sections are representative of three mice per group. Scale bar = 50 μm.

Supplementary Table S1. Differentially abundant bacterial phyla between WT and IgA KO mice.

Phylum	WT mice (%)*	IgA KO mice (%)	*p* value#
Desulfobacterota_G_459543	0	1.26 ± 0.93	< 0.05
Campylobacterota_A	0	0.81 ± 0.99	< 0.05

* Values are presented as the mean ± standard deviation (SD) of the relative abundances (%). Complete bacterial lists were shown in the Supplementary Table S1.csv file as a Supplementary Material. Each group was composed of four to five mice. # The relative abundances were compared using the Mann–Whitney *U* test.

Supplementary Table S2. Differentially abundant bacterial families between WT and IgA KO mice.

Family	WT mice (%)*	IgA KO mice (%)	*p* value#
Bacteroidales (UBA932)	0	8.99 ± 5.81	< 0.05
Bacteroidales (UBA11471)	0	2.46 ± 0.48	< 0.05
Rikenellaceae	0	1.30 ± 1.17	< 0.05
Desulfovibrionaceae	0	1.26 ± 0.93	< 0.05
Helicobacteraceae	0	0.81 ± 0.97	< 0.05
Tannerellaceae	2.21 ± 1.36	0	< 0.05
Burkholderiaceae_A_595427	0.62 ± 0.67	0	< 0.05

* Values are presented as the mean ± SD of the relative abundances (%). Complete bacterial lists were shown in the Supplementary Table S2.csv file as a Supplementary Material. Each group was composed of four to five mice. # The relative abundances were compared using the Mann–Whitney *U* test.

Supplementary Table S3. Differentially abundant bacterial species between WT and IgA KO mice.

Species	WT mice (%)*	IgA KO mice (%)	*p* value[#]
Cryptobacteroides sp009774765	0	4.20 ± 4.94	< 0.05
Muribaculaceae (UBA3263 sp001689615)	0	4.02 ± 2.30	< 0.05
Cryptobacteroides sp900546395	0	3.82 ± 1.29	< 0.05
Paramuribaculum intestinale	0	3.41 ± 1.09	< 0.05
Caccoplasma intestinavium	0	2.46 ± 0.48	< 0.05
Prevotella sp902776665	0	2.31 ± 0.45	< 0.05
Paramuribaculum sp900551515	0.07 ± 0.09	2.31 ± 1.76	< 0.05
Muribaculaceae (CAG-873 sp011959565)	0	2.27 ± 1.90	< 0.05
Bacteroides_H_857956 rodentium	0	2.25 ± 1.43	< 0.05
Paramuribaculum sp000431155	0	2.22 ± 1.08	< 0.05
Phocaeicola_A sartorii	0	2.07 ± 1.22	< 0.05
Helicobacter_C japonicus	0	0.80 ± 0.97	< 0.05
Lachnospiraceae (COE1 sp000403215)	4.13 ± 4.89	0	< 0.05
Bacteroides_H_857956 uniformis	2.99 ± 1.22	0	< 0.05
Bacteroides_H_857956 fragilis	1.19 ± 0.99	0	< 0.05
Parabacteroides_B_862066 goldsteinii	1.13 ± 0.71	0	< 0.05
Parabacteroides_B_862066 distasonis	1.08 ± 0.79	0	< 0.05
Ruminiclostridium_E siraeum	0.78 ± 0.64	0	< 0.05

* Values are presented as the mean ± SD of the relative abundances (%). Complete bacterial lists were shown in the Supplementary Table S3.csv file as a Supplementary Material. Each group was composed of four to five mice. [#] The relative abundances were compared using the Mann–Whitney *U* test.
