# Supplementary material for: IgA Deficiency Exacerbates an Animal Model of Multiple Sclerosis Induced with Theiler’s Virus
Source: Cells. 2026 Sep 15;15(18):1665. doi: 10.3390/cells15181665 (PMC13605160; doi:10.3390/cells15181665)
Supplement: Supplementary file 1 [file cells-15-01665-s001.zip › Supplementary Text S1.pdf]

Supplemental text S1. Donor DNA vector construction.

pflox-*Igha* ***M13F***, ***M13R***, LoxP, FRT, RE site
Exon (CDS/UTR) 5'arm/central arm/3'arm CRISPR Target

CACCTAAATTGTAAGCGTTAATATTTTGTTAAAATTCGCGTTAAATTTTTGTTAAATC
AGCTCATTTTTTAACCAATAGGCCGAAATCGGCAAAATCCCTTATAAATCAAAAGAA
TAGACCGAGATAGGGTTGAGTGTTGTTCCAGTTTGGAACAAGAGTCCACTATTAAA
GAACGTGGACTCCAACGTCAAAGGGCGAAAAACCGTCTATCAGGGCGATGGCCCA
CTACGTGAACCATCACCCTAATCAAGTTTTTTGGGGTCGAGGTGCCGTAAAGCACT
AAATCGGAACCCTAAAGGGAGCCCCCGATTTAGAGCTTGACGGGGAAAGCCGGCG
AACGTGGCGAGAAAGGAAGGGAAGAAAGCGAAAGGAGCGGGCGCTAGGGCGCTG
GCAAGTGTAGCGGTCACGCTGCGCGTAACCACCACACCCGCCGCGCTTAATGCGC
CGCTACAGGGCGCGTCCCATTCGCCATTCAGGCTGCGCAACTGTTGGGAAGGGCG
ATCGGTGCGGGCCTCTTCGCTATTACGCCAGCTGGCGAAAGGGGGATGTGCTGCA
AGGCGATTAAGTTGGGTAACGCCAGGGTTTTCCCAGTCACGACGTT***GTAAAACGAC***
GGCCAGTGAATTGTAATACGACTCACTATAGGGCGAATTGgagctccaccgcggtggcggccG
AAGTTCCTATAC*TTTCTAGA*GAATAGGAACTTCtgggatccacgtttCTGGGGTGAATTAG
CATGACTGGACTTATTCACAGTTCTAGCCTGAGCTTTGCTGGATTGTTAAACTCAGT
GCTGAGGTAACTGAAAAGACTTTGGATGAAATGTGAACCAACTCTTGTTTTATGGT
CCAGATTGTGGCCTCTGGTTTGCTTTGTGTGAATGGAGCCATGCAACCTAGTATCC
TATTCTCACACCCCCTCCTCCTTCCCTGATTACCTGGTGGAAATTCACATGTACCTG
TAAATGGTGCCTGCATGGCATGGACAGAGACACCACTCAGATTGAGTCCTCTCGTC
TTTGGGTCCACTGCTTGCTAAGTGGATGGGCAGTTGCTGGAGTGGTGGGTCTGAA
GCCAGAACAAGGCCAGTGTAAGGTCAAAATGGCCTTCTCTACCCCACACACAACCC
CCTGCCCCGCCAGTGTAACTTCCAAGCCAGCTCTTCCCCAACCACTCCTACCCCGT
GCTGGCTGATCTTCAGTCTCAGGTTGGCCACCCCTGCCCAGACCCACCAGTTCTGG
TCCTCCTAACCCTGGGACTGAGAACATAGCTCTATCCACTGCCCAAACAGGAGTGG
GGCTCAGAAATCTGGAGCGCTAGACTGCTCAGGGGTGTAGTCATGTTTACAAACGC
ACAGTATGTGCAAAGCCCTGCTGGAGTCATTTGGCTAGATCCTTGTGAAAGACTAC
CTGCAGGTCATGTTCAAAGTCTATACAGCCAGAACTGTTGGTCAGCTCCGACTGCA
GGTACACGATGCAGCAGCTGTGTGATACTGGGCTAGGTTCTCCTGTATAAAGAAGA
GAAAGGCATGGTCCTTTTTCCTGAAATTGTCTCGAGATGGGCAGTGTGAAGACTAC
TATCTCATGCATGTTTTATGTTCCAGAGTCTGCGAGAAATCCCACCATCTACCCACT
GACACTCCCACGAGCTCTGTCAAGTGACCCAGTGATAATCGGCTGCCTGATTCACG
ATTACTTCCCTTCCGGCACGATGAATGTGACCTGGGGAAAGAGTGGGAAGGATATA
ACCACCGTAAACTTCCCACCTGCCCTGGCCTCTGGGGGAGGGTACACCATGAGCA

GCCAGTTGACCCTGCCAGCTGTCGAGTGCCCAGAAGGAGAATCCGTGAAATGTTC
CGTGCAACATGACTCTAACGCCGTCCAAGAATTGGATGTGAAGTGCTCTGGTAAAG
AACGTTAGGGGGTCAGCTGGGGGTGGATAAGTTCTACCTTATCTAGATCCATATAT
CCCTCTGAGGCACACCCTCACAGGGACCCTCAGAAACCTCCCaaacATAACTTCGTA
TA*GCATACAT*TATACGAAGTTATggcgcgccATGGGGGTGGGGGAAGGGAAGCGTAAA
CAGGCCATAAGGAACTGAGGCCTCAGAACATCCAGAAAAGGGGACAGCAAAGGAG
AAAAGGAGAATATACTGATTTGCTAGGACTTCTCTGTTACAGGTCCTCCTCCTCCTT
GTCCTCCTTGTCCTCCTTCCTGCCATCCCAGCCTGTCACTGCAGCGGCCAGCTCTT
GAGGACCTGCTCCTGGGTTCAGATGCCAGCCTCACATGTACTCTGAATGGCCTGAG
AAATCCTGAGGGAGCTGTCTTCACCTGGGAGCCCTCCACTGGGAAGGATGCAGTG
CAGAAGAAAGCTGTGCAGAATTCCTGCGGCTGCTACAGTGTGTCCAGCGTCCTGC
CTGGCTGTGCTGAGCGCTGGAACAGTGGCGCATCATTCAAGTGCACAGTTACCCAT
CCTGAGTCTGACACCTTAACTGGCACAATTGCCAAAATCACAGGTGAGCCCAGATG
CATACCCGGGACATTGTATGACGTTCCCTGCTCACATGCCTGCTTTCTTCCTATAAT
ACAGATGCTCAACTAACTGCTCCTGTCCTTATATCACAGAGGGAAATTGGAGCTATC
TGAGGAACTGCCCAGAAGGGAAGGGCAGAGGGGTCTTGCTCTCCTTGTCTGAGCC
ATAACTCTTCTTTCTACCTTCCAGTGAACACCTTCCCACCCCAGGTCCACCTGCTAC
CGCCGCCGTCGGAGGAGCTGGCCCTGAATGAGCTCGTGTCCCTGACATGCCTGGT
GCGAGCTTTCAACCCTAAAGAAGTGCTGGTGCGATGGCTGCATGGAAATGAGGAG
CTGTCCCCAGAAAGCTACCTAGTGTTTGAGCCCCTAAAGGAGCCAGGCGAGGGAG
CCACCACCTACCTGGTGACAAGCGTGTTGCGTGTATCAGCTGAACTCTGGAAACAG
GGTGACCAGTACTCCTGCATGGTGGGCCACGAGGCCTTGCCCATGAACTTCACCC
AGAAGACCATCGACCGTCTGTCGGGTAAACCCACCAATGTCAGCGTGTCTGTGATC
ATGTCAGAGGGAGATGGCATCTGCTACTGAGCCACCCTGCCTGTCCCTACTCCTGA
AATAAACTCTGTGCTCATCCAAAGTATCCCTGCACTTCCATCCAGTGCCTGTCCATC
ATCCTTAGGGTCTACAGAACACAGGGAGGGGTCAGGGCCCAGGGAGGGAGAAACA
CCACCACCTAAGCTTGTAAGGCCTCGGAGACTTTGAAGACCCTTCATGCCTAGAGT
ATATGTGTATGCATGTCTATGTATTGGTGAGTGCTTATGGATGGGTCCATGTGCCTT
CATGTGTGTACCTGTGTGCAGCAAGAGAGGAGTGAGTTTGTGTGCACCTATGGTTT
ATAGGTATGTATATGGGTATAAGTACATATGTGGACACACTATATCTATGTCCCTGTG
AGCAAGTGTGTGCCTGTGCATACCTATGAGCATGCATATATCTATGTGTGTTCATGT
TGGTGGAAACATGAGAGTGTGTGCACCTGCCCTGTGAATGTGCAATGAGTGAATG
GGAAGTAAGAATAAACTGGGGATGAAATCCAAGGCGAGCAGACTCAGCCAAGAAG
GCAAAGCTCATGGGAGAGTCCCCAGGGCAGTGGGTTGCTGCACAAGTACTCTGAG
TGGTGGGTGGTGTGGCTGTTTATTAGACCGCAGCTTCTAACCCAAACAAGACAGGG
ATAGATGGAGCAAAAGGGTAGCACCATCAAGGCTGGTGCCGTGTGGGGGTCACAA

TAGAGATGATGGTATGAGAATGAAGGGATGAACACAGGGGCACTGGGAAGAAGAT
GCCACGCACCCTCAGTAGGCCATCCCTGAATGGAAACATGCATTCAGGGTGACCAC
TGACTCATGAGAAAGACATATAGCATGGCACAGACAACCCTCAGCAGCACCCTGCT
CTTCACACATGCACATGGGCATACACCCTGTTCTGCATACATGCACATGGGCATACA
TGCACAGACACTCACATTTGCACACTCACACAGACCTGAACCCTGGAGGAAGATGA
AGTTCACAGCCCACACCCAGCTTCTGGAAAGCAAGGGGAGTCCCCTCCAAGCCCC
CGGGAGAATTTCCAGGCCCGACACTGACAACTACACACAATGGACAAGGGCATAG
GCCCCTCCAGGTGCCCCCTTTCTAACCTAAGTCTCCCTTCCCATCCTGAGCCCAAG
TATCACAAACCAGAAGCAGGATAAGAGGCAAATCTGGGAAGAAGCAGGAGCAGGC
CATGGAGACCACTCCCACACAGGCCTCGGCCTCCTCCATGGGCACCCCTAGAACA
CAAAGTTCTACAACCAGGTATACTTGGAGCCCAGGTGTCCACAAGAGGGCAGTGG
GGGCCCTGCTCCTGTCTCACAGGCCTTCAGAACTTCGGGGGGGGGGGGAGGAG
TTGCTGGTTTCTAGCAACTTCCCTCTGCTCTCAGAGCAAGAGTGGAGTAAAGGCCC
ATGCATGCCCTGGATACTCTCCAGCTTGAAAGTCCAGTTCCCTGAGAATAGCAAGA
GCAACAGACCAGCTTGGGAAACCTGACTCAGAAAAGTACCATAGCCACCCATGGA
ATCCAAGGCCTGGCCCATGACAGAGAATCCTAGTAAGGAGGAGTCAATAGGTGAAA
GACTGAATCCCCACAGGGAGAGGGGGCAGAGAAGCTGGAGAATCACGATAGTCGG
GAAAGATGATGAGTGAGCGGCTGGAGGGTAATGGATGGCTGGCTACAAGGATGAA
CGATGGGGATGATGGATGGATTGATGGATGGCTGCAGTGACAGAAATACGAGTGG
TTGACTATCAGTACACATTTCCTACTGCCACTTAAAATGAAACTCTGTGCGCAGAGA
ACCTGAGGACAGCAGGGTTGGCTCTACTGGGGGTCTTTACTGTTGCTTAACCCCCA
GTCACAGCTCTTAAGAATGCAACAGAGTCCTCTGACCACATGTCCAGGCCTTGGTA
GTCTAACTTTGGCCCAGAAATCACATTGGCCTGTATCTGTTTGAAGGTGTCTGATCT
TACACAACCCTAGTACCCAAGCCAAGATTAGCCTAAGAACTGGGGTTTAGGGAGGT
ATTGCTAAATATCTAGAAAGGACTGAGACCTAAGGAGCCTTGGGTTCAAACTCTTC
TCAGAGTTTGACAGAAACAACTCTCTTACCATGACAGGTCTCTAATTAAGGTTTCCC
TAAGAGAGAGAGATGGGTCTTGGGGCCATCTCAAGAACTGCTGGGATTCCAGTCC
CTTACCTGGGACCCTGCCTCCTCATTATCCCTGAACAAGTCCCTTCCCGGGAATAA
GGGACCCCTACCACCACTGTCCCCAAAGCCCAAGGAGGCATAGAAGCAGAAACCT
TAAGTCTGAGCCCCCATCCCCATGGGTTTGGATCTAGAAGGTCATCTATGTCTTACA
G**AACGTCAAGAGCCACTTTCCTATGTGCTACTGGACCAGTCAGAAGACATCCTGGA**
GGAAGAGGCCCCAGGTGCCAGCCTGTGGCCCACCACTGTGACCTTCCTCACCCTC
TTCCTACTGAGCTTGTTCTACAGCACAGCACTCACTGTTACAACTGTTCGAGGCCC
GTTTGGCAGCAAAGAGGTCCCCCAGTACTGA*CCAGGAGCCAGCCACAGGTGGCAG*
TCATGGAGATGGTGGGGTACAGGGTGGGGGCAGGGGCACTCAGGATTTACTAGGC
TCCTCCATATGTGGCCTTGCAACCCTTCACAGACACTAAGGAAGTGGACGTAAATG

ATGGCGGCACTTCCTGGGTTTAACCCAATCTTCCAGAAGCCAGGCTGGGACCTCAG
CAAAAGGGAAGACATAACCCAACTCCAGGATGCCCTGTGGCTGATCACTCTTGTCT
*TG*cgcgccaattcgatatcaagctATAACTTCGTATA*GCATACAT*TATACGAAGTTATgcggcc*GC*
GGGGATGAAGGTTGGAGAAAGGCCTCCACGTTTCAGAGCTAAGACCGACGTGACA
ACTTCCCAGCCCACTCACAGACTTTCCTGAGCAATAAATTGATGCAAAACCACAAA
TTCCTACTCTCAAAAACAAACATTAACAAAGGATTGGGGGAGGGGGTCAGGGGTA
GTATGGTGGCGTTGGGCAGGGTAAACTCAGGGTACCCATTGTCTAATGTCTGAGAC
ATAACTTGAACATATGTGTAGCTGCAGCCAAAGATGAACAAGTGATGGTATTTGTGT
CCTCTTCAGACCCGATACCAGGTCATTAAGCTTGAGATCTGGACCTGATTTCTAAAC
ATTTGGCCTCTGTGAGCACCCTTGGCAAATACTGAACAGCAAACCCTGGTCCTGGC
TGTGAACCTTGGTCCTGATCACTGAGCCCTATATTGGTAACTGAACCGTGATCCTG
ATCTCTGACCTCAGTCATGGTCATGAGGCCCTGGTCCTGGCCACTCATTCTAATAA
CTGGTCCCTAGTCCTGAGCCCTGAACCTTGGTCCAGGTCACTGAATCCTAGTCATG
ATCACTGGGTTTGATCCTCATTACTGAGCCTAGTCTTGATCACCGATGACTGGTTTT
GATCATAGCAATTAACCTGATCACTAGTCCCCGTTCCTCATCACTGGGCCATGATAC
TGGTCACTGGGTCCTGATCCTGATCACTAAATCCTGTTTCTAAACAATGTGTAGTGG
AATGTATAGTGAAGCCTTTGTGTCTGGCTCTGGGTGAAATGTCTCAGCAGAGCCTT
TGCTAGGTTTGGGTTAATCAGTTGGGGCTGAGAAATGTTTTTGAGGCTGTTTGAAC
TTCAAAAGAAGAAATGTCTCCCTGGACAATCTGCACATTTGCAGCTGCGCAAACCT
TCATCCTAAAACTTAACTCCTGGCAAACTTAGAATTCTTACTTTTAATAATGGCTAG
CCATGGTTGAAAGGGACTGAGATGTCTGTGGGTGGGTGGAACCTTTCCCAGCTCC
AAGTAACTCTGTATACTGTTTGAATAAAGTAACTGAAGTGAGCTAGCTGGGGTCAA
*TCTTCTTTCCAAGGAGAATAAAGCCCTCCGCTCCTCCAGAAAATGAAGGCTTA*GCT
CCTTGGTTAGCTTCTCTCTCTACTGCGGCACCTACAACCAACTCAGCAGTCCTAGG
TTCCTGTCACCAGATCCAGTCCTGATAGCTAAGTGTCAATCCTCGTCACTAAGGCC
TGATCCTTAGCAATATGCCTGGGGTCTGATAATCAggccgcgaattcGAAGTTCCTATAC*T*
*TTCTAGA*GAATAGGAACTTCtcgagggggggcccggtaccCAGCTTTTGTTCCCTTTAGTGAG
GGTTAATTGCGCGCTTGGCGTAATCATG***GTCATAGCTGTTTCCTG***TGTGAAATTGTT
ATCCGCTCACAATTCCACACAACATACGAGCCGGAAGCATAAAGTGTAAAGCCTGG
GGTGCCTAATGAGTGAGCTAACTCACATTAATTGCGTTGCGCTCACTGCCCGCTTT
CCAGTCGGGAAACCTGTCGTGCCAGCTGCATTAATGAATCGGCCAACGCGCGGGG
AGAGGCGGTTTGCGTATTGGGCGCTCTTCCGCTTCCTCGCTCACTGACTCGCTGCG
CTCGGTCGTTCGGCTGCGGCGAGCGGTATCAGCTCACTCAAAGGCGGTAATACGG
TTATCCACAGAATCAGGGGATAACGCAGGAAAGAACATGTGAGCAAAAGGCCAGC
AAAAGGCCAGGAACCGTAAAAAGGCCGCGTTGCTGGCGTTTTTCCATAGGCTCCG
CCCCCCTGACGAGCATCACAAAAATCGACGCTCAAGTCAGAGGTGGCGAAACCCG

ACAGGACTATAAAGATACCAGGCGTTTCCCCCTGGAAGCTCCCTCGTGCGCTCTCC
TGTTCCGACCCTGCCGCTTACCGGATACCTGTCCGCCTTTCTCCCTTCGGGAAGCG
TGGCGCTTTCTCATAGCTCACGCTGTAGGTATCTCAGTTCGGTGTAGGTCGTTCGC
TCCAAGCTGGGCTGTGTGCACGAACCCCCCGTTCAGCCCGACCGCTGCGCCTTAT
CCGGTAACTATCGTCTTGAGTCCAACCCGGTAAGACACGACTTATCGCCACTGGCA
GCAGCCACTGGTAACAGGATTAGCAGAGCGAGGTATGTAGGCGGTGCTACAGAGT
TCTTGAAGTGGTGGCCTAACTACGGCTACACTAGAAGGACAGTATTTGGTATCTGC
GCTCTGCTGAAGCCAGTTACCTTCGGAAAAAGAGTTGGTAGCTCTTGATCCGGCAA
ACAAACCACCGCTGGTAGCGGTGGTTTTTTTGTTTGCAAGCAGCAGATTACGCGCA
GAAAAAAAGGATCTCAAGAAGATCCTTTGATCTTTTCTACGGGGTCTGACGCTCAG
TGGAACGAAAACTCACGTTAAGGGATTTTGGTCATGAGATTATCAAAAAGGATCTT
CACCTAGATCCTTTTAAATTAAAAATGAAGTTTTAAATCAATCTAAAGTATATATGAG
TAAACTTGGTCTGACAGTTACCAATGCTTAATCAGTGAGGCACCTATCTCAGCGATC
TGTCTATTTCGTTCATCCATAGTTGCCTGACTCCCCGTCGTGTAGATAACTACGATA
CGGGAGGGCTTACCATCTGGCCCCAGTGCTGCAATGATACCGCGAGACCCACGCT
CACCGGCTCCAGATTTATCAGCAATAAACCAGCCAGCCGGAAGGGCCGAGCGCAG
AAGTGGTCCTGCAACTTTATCCGCCTCCATCCAGTCTATTAATTGTTGCCGGGAAG
CTAGAGTAAGTAGTTCGCCAGTTAATAGTTTGCGCAACGTTGTTGCCATTGCTACA
GGCATCGTGGTGTCACGCTCGTCGTTTGGTATGGCTTCATTCAGCTCCGGTTCCCA
ACGATCAAGGCGAGTTACATGATCCCCCATGTTGTGCAAAAAAGCGGTTAGCTCCT
TCGGTCCTCCGATCGTTGTCAGAAGTAAGTTGGCCGCAGTGTTATCACTCATGGTT
ATGGCAGCACTGCATAATTCTCTTACTGTCATGCCATCCGTAAGATGCTTTTCTGTG
ACTGGTGAGTACTCAACCAAGTCATTCTGAGAATAGTGTATGCGGCGACCGAGTTG
CTCTTGCCCGGCGTCAATACGGGATAATACCGCGCCACATAGCAGAACTTTAAAAG
TGCTCATCATTGGAAAACGTTCTTCGGGGCGAAAACTCTCAAGGATCTTACCGCTG
TTGAGATCCAGTTCGATGTAACCCACTCGTGCACCCAACTGATCTTCAGCATCTTTT
ACTTTCACCAGCGTTTCTGGGTGAGCAAAAACAGGAAGGCAAAATGCCGCAAAAA
AGGGAATAAGGGCGACACGGAAATGTTGAATACTCATACTCTTCCTTTTTCAATATT
ATTGAAGCATTTATCAGGGTTATTGTCTCATGAGCGGATACATATTTGAATGTATTTA
GAAAAATAAACAAATAGGGGTTCCGCGCACATTTCCCCGAAAAGTGC
